# Cold-Water Corals and Anthropogenic Impacts in La Fonera Submarine Canyon Head, Northwestern Mediterranean Sea

**DOI:** 10.1371/journal.pone.0155729

**Published:** 2016-05-16

**Authors:** Galderic Lastras, Miquel Canals, Enric Ballesteros, Josep-Maria Gili, Anna Sanchez-Vidal

**Affiliations:** 1 Grup de Recerca Consolidat en Geociències Marines, Universitat de Barcelona, Barcelona, Spain; 2 Centre d’Estudis Avançats de Blanes, Consejo Superior de Investigaciones Científicas, Blanes, Spain; 3 Institut de Ciències del Mar, Centre Mediterrani d'Investigacions Marines i Ambientals, Consejo Superior de Investigaciones Científicas, Barcelona, Spain; Università di Genova, ITALY

## Abstract

We assess the occurrence and extent of cold-water coral (CWC) species *Madrepora oculata* and *Dendrophyllia cornigera*, as well as gorgonian red coral *Corallium rubrum*, in La Fonera canyon head (Northwestern Mediterranean Sea), as well as human impacts taking place in their habitats. Occurrence is assessed based on Remotely Operated Vehicle (ROV) video imaging. Terrain classification techniques are applied to high-resolution swath bathymetric data to obtain semi-automatic interpretative maps to identify the relationship between coral distribution patterns and canyon environments. A total of 21 ROV immersions were carried out in different canyon environments at depths ranging between 79 and 401 m. Large, healthy colonies of *M*. *oculata* occur on abrupt, protected, often overhanging, rocky sections of the canyon walls, especially in Illa Negra branch. *D*. *cornigera* is sparser and evenly distributed at depth, on relatively low sloping areas, in rocky but also partially sedimented areas. *C*. *rubrum* is most frequent between 100 and 160 m on highly sloping rocky areas. The probable extent of CWC habitats is quantified by applying a maximum entropy model to predict habitat suitability: 0.36 km^2^ yield *M*. *oculata* occurrence probabilities over 70%. Similar predictive models have been produced for *D*. *cornigera* and *C*. *rubrum*. All ROV transects document either the presence of litter on the seafloor or pervasive trawling marks. Nets and longlines are imaged entangled on coral colonies. Coral rubble is observed at the foot of impacted colonies. Some colonies are partially covered by sediment that could be the result of the resuspension generated by bottom trawling on neighbouring fishing grounds, which has been demonstrated to be responsible of daily increases in sediment fluxes within the canyon. The characteristics of the CWC community in La Fonera canyon are indicative that it withstands high environmental stress of both natural and human origin.

## Introduction

Reef-forming cold-water corals (CWC) provide ecological niches and substrate that harbour a large variety of other species acting both as refuges for prey as well as nursery areas [1]. They are the basis of unique ecosystems on the World’s continental margins [2, 3, 4], forming kilometres-long belts of living reefs and massive mound structures in the Atlantic Ocean [5, 6, 7, 8]. The term “cold-water corals” encompasses stony corals (Scleractinia), true soft corals (Octocorallia, including red corals), black corals (Antipatharia), and calcifying lace corals (Stylasteridae); being scleractinian white corals *Lophelia pertusa* and *Madrepora oculata* the predominant reef-forming species [9], often accompanied by *Dendrophyllia cornigera* [2].

These sessile suspension feeders prefer hard substrates within high energetic hydrodynamic environments that prevent deposition of sediment and where nutrient-rich waters stimulate food supply [9, 10] in a temperature range of 4–13°C and a salinity range of 32–38.8‰ [1]. Until recently, most cold-water scleractinian coral specimens collected in the Mediterranean Sea were dead, but few living scattered patches have been reported setting on steep cliffs in escarpments, canyon walls and rocky outcrops, as well as forming mounds [11, 12, 13, 14, 15, 16, 17, 18, 19, 20, 21, 22, 23]. They occur at depths ranging between 150 m in the Gibraltar Strait to more than 700 m in the Ionian Sea [15, 17]. Nevertheless, the true expanse of such communities in terms of both lateral extension and bathymetric range is poorly constrained and biased by the low number of observations [19]. Access to advanced instrumentation to explore deep-water environments such as manned and Remotely Operated Vehicles (ROVs) is progressively revealing the scale and abundance of CWC colonies, although the rough topography of coral-prone areas such as submarine canyons makes difficult an accurate positioning and endangers the operation of underwater vehicles.

The acquisition of high-resolution multibeam data for identifying potential locations and subsequent ground-truthing by in situ direct observations is a primary step for improving the understanding of CWC ecosystems [9]. In addition, these data are the basis for habitat characterization and allow the automatic generation of predictive maps of benthic habitats. Such a modelling approach has been applied at a global scale [24, 25] and at a higher resolution to delve into CWC provinces such as Porcupine Bank [26], Gibraltar Strait [27] and the Northern Ionian Sea [28].

CWC ecosystems are slow-growing and fragile, and thus are considered Vulnerable Marine Ecosystems (VME). On a global scale, the effects of ocean acidification on CWC are considered to be potentially catastrophic [3, 29]. Local threats to CWC include anthropogenic activities like bottom trawling, hydrocarbon and mineral exploration and exploitation, and cable and pipeline placement [2, 9]. Long-lived gorgonian red coral *Corallium rubrum* occasionally found in association with CWC, reported on rocky bottoms at depths spanning between 7 and 300 m in the Mediterranean Sea but observed alive at 600–800 m in the Strait of Sicily [30], has in addition been heavily harvested for jewellery [31, 32]. A number of countries and regional bodies have developed regulations and measures to locally protect these ecosystems, for example by establishing Marine Protected Areas (MPA) and closing CWC habitats to bottom fishing, but first and foremost important is locating them and mapping their extent.

CWC have been reported at different locations in the Northwestern Mediterranean Sea. Mainly *M*. *oculata* but also *L*. *pertusa*, *D*. *cornigera* and *Desmophyllum cristagalli* have been observed at the head and upper flanks of Cap de Creus canyon [20]. Whereas the latter appear mostly as isolated colonies, large healthy communities of *M*. *oculata* lay over some 4.7 km^2^ of hard substrate patches at depths of 200–300 m [20, 33]. *C*. *rubrum* has been equally reported in Cap de Creus down to 230 m [34]. Two white coral species have also been observed at Lacaze-Duthiers canyon head and western flank, with *L*. *pertusa* being found deeper (350–541 m) than *M*. *oculata* (246–531 m), whereas in the Cassidaigne canyon, east of Marseille, only *M*. *oculata* has been reported on its western flank, from 200 m down to 1000 m, co-occurring with *C*. *rubrum* colonies in the depth range of 200 to 325 m [11, 12, 22, 23, 35].

**Fig 1:**
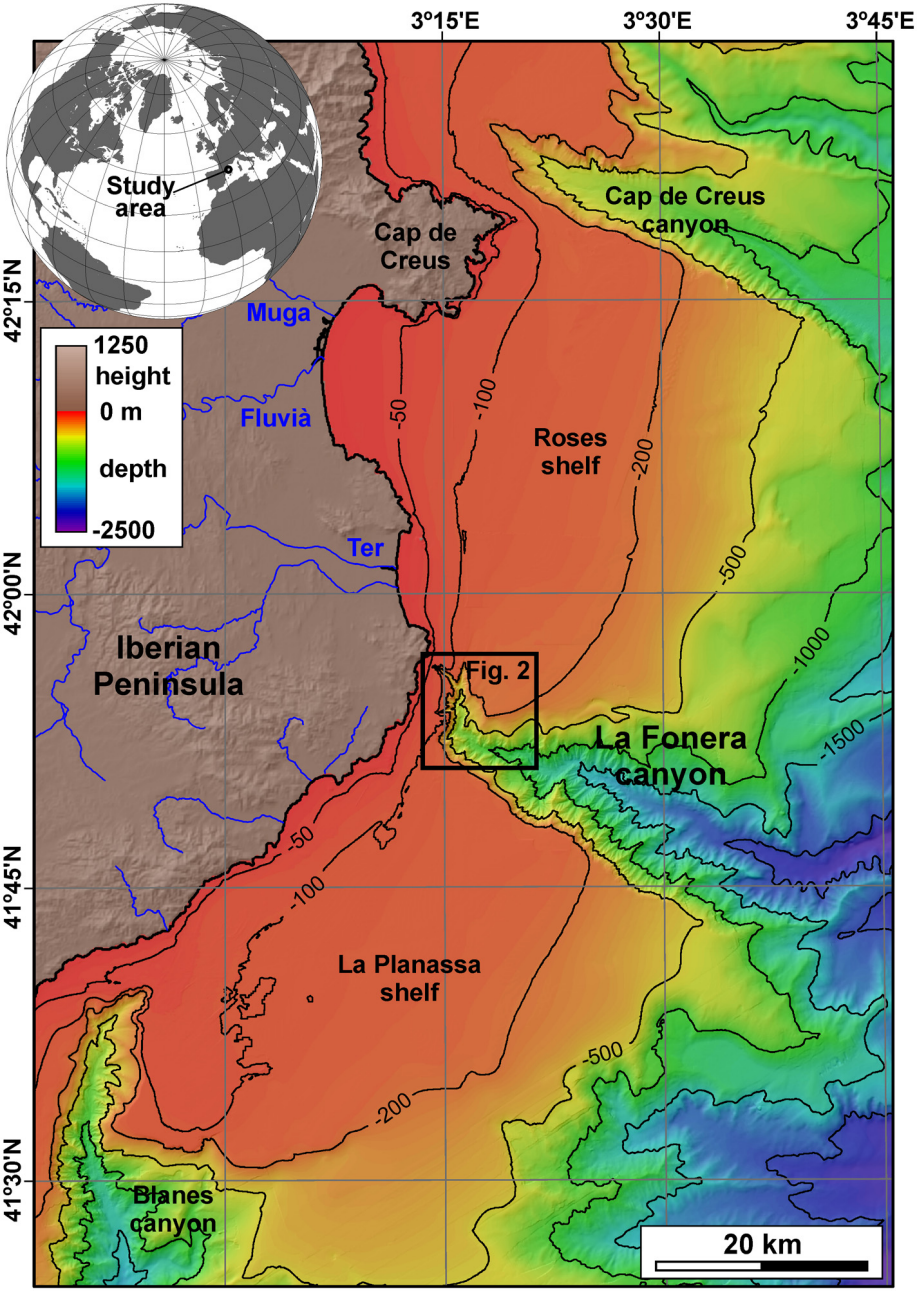
Topo-bathymetric map of the northern Catalan margin. Locations of the study area and the main rivers draining into the margin are indicated.

**Fig 2:**
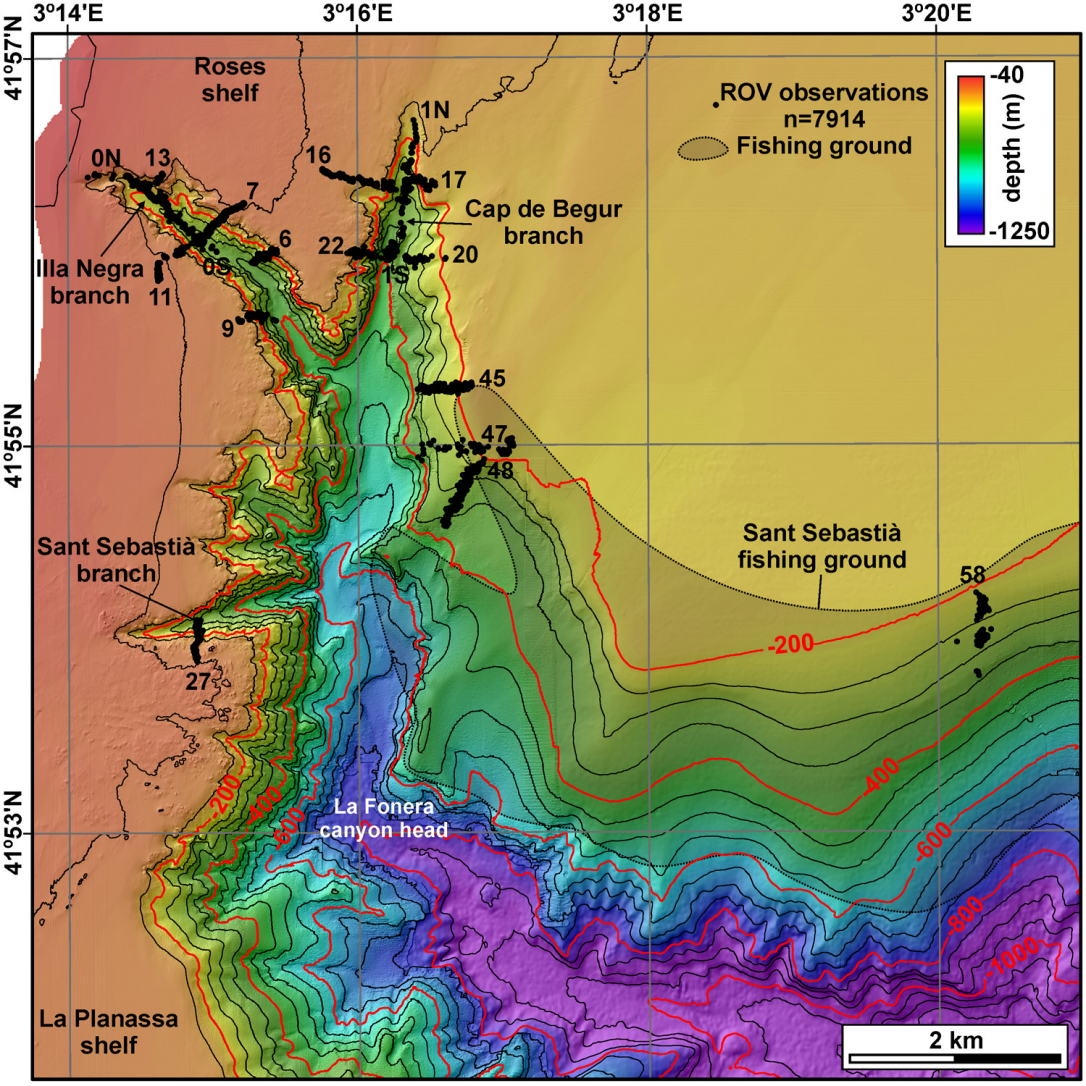
Bathymetric map of La Fonera canyon head and its neighbouring continental shelf and upper slope. Locations of the three main canyon branches (Cap de Begur, Illa Negra and Sant Sebastià), the ROV observations used in this study and the Sant Sebastià fishing ground are indicated.

Thus, since living CWC ecosystems in the Mediterranean Sea have mostly been reported from submarine canyons, and following first positive reports [36], La Fonera canyon was a good candidate to host extensive communities. This canyon is located in the Northwestern Mediterranean Sea immediately south of Cap de Creus and Lacaze-Duthiers canyons, where coral communities have already been described [20, 22]. La Fonera submarine canyon dissects the North Catalan margin along 110 km from its head down to 2550 m water depth in a roughly NW-SE direction [37]. From rim to rim, it has a width of 7.1 km at 1000 m axial depth [38], and its walls, often carved by pervasive networks of gullies, have gradients exceeding 25°. Together with Cap de Creus canyon to the north and Blanes canyon to the south, they make up the main pathways connecting the inner shelf and the deep sea in the Catalan margin (Fig 1). The head of La Fonera canyon incises 28 km into the continental shelf following a WNW-ESE trending course, with a N-S oriented shallowest part [36, 37] (Fig 2), and represents the limit between Roses continental shelf to the north and La Planassa continental shelf to the south [39] (Fig 1). At water depths above 800 m, the head is formed by a network of three main and many minor branches and gullies [36]. The three main branches are (1) Cap de Begur branch, which runs in a N-S direction and has its head tip located at 135 m water depth; (2) Illa Negra branch, which is NW-SE oriented and has its head tip located at 60 m water depth at a distance of barely 800 m from the coastline; and (3) Sant Sebastià branch, which is W-E oriented and has its tip located at 90 m water depth (Fig 2).

The floor of the narrow shelf located between the canyon head and the coastline, at water depths less than 125 m, is characterised by irregular to sub-rounded rocky outcrops, which also comprise the rough terrain of the highly-sloping walls of Illa Negra and Sant Sebastià branches, as well as the western wall of Cap de Begur branch [36]. These outcrops are most probably formed by Hercynian granitoids and Cambro-Ordovician schists and limestones [40]. In contrast, the Roses shelf and upper slope have a rather sedimentary nature [36, 39]. In the eastern wall of Cap de Begur branch, layered sedimentary strata outcrop at water depths less than 700 m among a smoother canyon wall [36]. The limit between the inner and outer Roses shelf, at 125 m water depth, is marked by the Roses shelf channel, a 1500 m wide, 15 m deep channel-like depression that extends along the entire Roses shelf from offshore Cap de Creus and into Cap de Begur branch in La Fonera canyon [39]. La Fonera canyon floor is relatively flat and smooth, and has a width ranging between 300 and 400 m.

The North Catalan margin as a whole is fed by sediment input from medium-to-small rivers and ephemeral streams [41]. From north to south, Muga, Fluvià and Ter rivers open to the Roses shelf (Fig 1), with mean water discharges of 3.0, 9.4 and 12.1 m^3^·s^-1^, respectively, and predicted sediment fluxes of 80·10^3^, 149·10^3^ and 176·10^3^ t·yr^-1^, respectively [41]. Dominant waves over the Roses shelf mainly come from the NNW and N, whereas south of La Fonera canyon over the La Planassa shelf they mostly come from the NNE and SW, and the most energetic ones trigger alongshore southwards drift able to transport large volumes of sediment [39].

The general circulation pattern in the study area is dominated by the Northern Current, a cyclonic mesoscale current flowing south-westwards over the outer shelf and canyon, but circulation within the canyon is constrained by the local bathymetry and the canyon shape. Bottom currents in the canyon head are usually below 20 cm·s^-1^, as measured at 470 m water depth in the canyon axis, with current directions mainly oriented up- and down-canyon along the axis direction [42, 43]. Near-bottom downward total mass fluxes at 470 m amount a mean of 28.8 g·m^−2^ day^−1^, peaking up at >94 g·m^−2^ day^−1^, with 2.4% of organic matter [42, 43, 44]. Turbidity time series at 470 m show suspended matter concentrations ranging between 0.4 and 1.4 mg·l^−1^, with high turbidity events reaching values of 40 mg·l^-1^ and lasting 1 to 6 hours associated with sediment gravity flows triggered by trawling activities [42].

Exceptional oceanographic events are storms and dense-shelf water cascading, during which La Fonera canyon is able to funnel suspended sediment and associated nutrients and pollutants [38, 42, 45, 46]. Concurrent dense shelf water cascading events and eastern storms may generate near-bottom current speed peaks of >50 cm·s^-1^, suspended sediment concentrations over 6 mg·l^-1^ and instantaneous sediment fluxes >2.4 g·m^−2^ s^−1^ at 370 m water depth in Illa Negra branch as measured in situ in March 2007 [46]. These values increase at 1200 m water depth, since sediment enters the canyon not only through its head but also across the canyon walls. Near-bottom temperature at 370 m water depth in Illa Negra branch is in the 13.2–13.4°C range, with values slightly below 12.6°C during cascading events [46].

As stated above, bottom trawling generates sharp increases in mass fluxes within the canyon [42, 44, 47]. Bottom trawling takes place on the shelf and along both canyon flanks, with special intensity in the northern rim and canyon wall along the Sant Sebastià fishing ground down to water depths of about 750 m (Fig 2). A mooring placed at 980 m depth within a tributary valley on the northern canyon flank revealed a daily occurrence of sediment transport events linked to the passage of the trawling fleet upslope the site, with maximum downslope velocities of up to 38 cm·s^−1^ and concentrations of up to 236 mg·l^−1^ close to the bottom [43, 48]. Thus, bottom trawling-generated flows are equivalent in the long term to natural small-scale landslides [48], and are responsible of widespread erosion of recent sediment [49] and large-scale changes in the seascape of the region [48]. Bottom trawling can also contribute to the development of slope nepheloid layers, and thus its effects can propagate away from fishing grounds [50].

La Fonera canyon shows the highest mean concentrations of litter on the deep-sea floor ever reported, with a mean exceeding 15,000 items·km^-2^ calculated after ROV observations [51]. Litter mostly concentrates on the canyon floor at water depths exceeding 1000 m, whereas canyon walls seem to be less impacted.

In this paper, we present a systematic ROV exploration of different canyon environments within La Fonera canyon head aiming at detecting CWC communities and quantifying their extent by applying predictive habitat mapping techniques based on high-resolution swath bathymetry data. In addition, we describe the anthropogenic impacts that these habitats are currently experiencing. The results of this study may represent a first step for establishing protection measures in an area extremely pressured by bottom trawling.

## Materials and Methods

### Bathymetric data

Swath bathymetry data from La Fonera canyon head and neighbouring continental shelf were obtained during the “Euroleón” cruise onboard *B*.*I*.*O*. *Hespérides* in October 2007 using a Simrad EM-1002S multibeam echosounder. It works at a frequency of 95 kHz with 111 beams per ping, and was operated in equidistant mode, that is, with swath width independent of water depth and fixed to 500 m. Data were logged using Kongsberg's Seafloor Information System (SIS) and processed using Caris Hips and Sips software, yielding a bathymetric grid with a resolution of 4 m (Fig 2). Derivative backscatter data were processed using SwathEd software developed by the Ocean Mapping Group, University of New Brunswick, yielding a backscatter grid with a resolution of 20 m. A detailed morphological description of La Fonera canyon head based on these data was published by [36].

### Benthic terrain modelling

**Fig 3:**
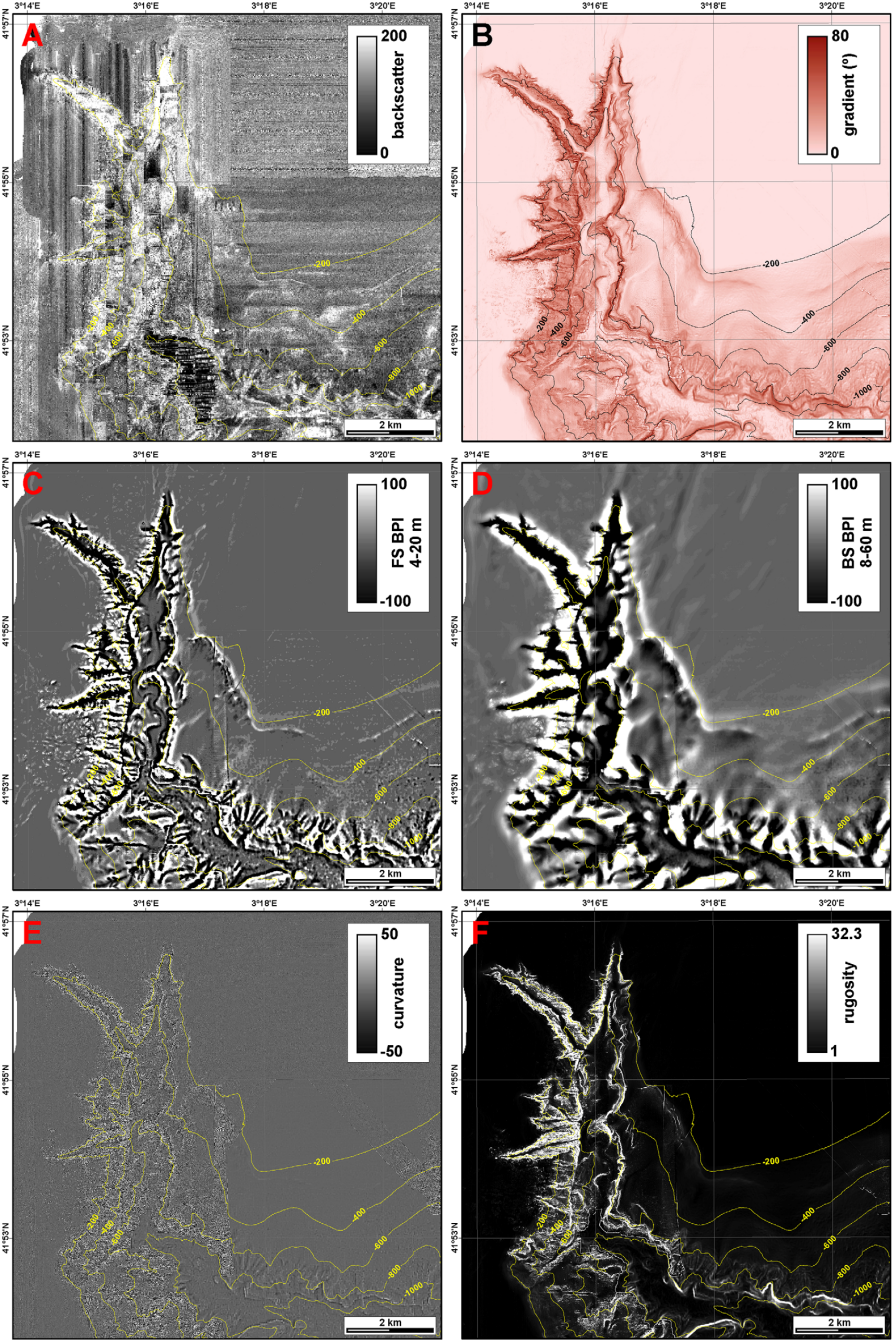
Bathymetry-derived parameters and backscatter of the study area. A: Backscatter data, represented in dimensionless digital number. B: Seafloor gradient. C: Fine-scale bathymetric positioning index (FS BPI). D: Broad-scale bathymetric positioning index (BS BPI). E: Seafloor curvature. F: Seafloor rugosity.

Terrain classification techniques were applied to bathymetric data in order to obtain a semi-automatic interpretative map, as a basis to assess the relationship between coral distribution patterns and the morphological seabed expression at various locations. The benthic terrain model tool (BTM) creates a terrain model that ascribes each location into a zone describing the structure and seabed complexity that characterizes the physical environment, and is usually based upon two primary data sets: bathymetry (Fig 2) and backscatter (Fig 3A). From the first of them, other bathymetric parameters such as seafloor gradient (Fig 3B), bathymetric position index (BPI) (Fig 3C and 3D), curvature (Fig 3E) and rugosity (Fig 3F) are derived, and are later combined through a set of standard algorithms to classify the benthic landscape and perform maximum entropy prediction of habitat suitability.

Curvature is calculated as the second derivative of the surface, and rugosity is defined as the ratio of surface area to planar area, thus measuring terrain complexity indicative of areas with high biodiversity potential. BPI is a second-order derivative of swath bathymetry, and indicates positive and negative terrain features [52], measuring where a given location is (in terms of depth) relative to the locations surrounding it. It calculates for each cell the mean depth around a minor given radius through a neighbourhood function and compares it to the mean depth around a major given radius. Positive cell values denote features that are higher than the surroundings and, inversely, negative values indicate features lower than the surroundings. The first ones may be associated to ridges or edges such as canyon rims, whereas the others relate to valleys or depressions. BPI rasters can be built at different scales with the aim to identify small or large scale features on the seascape.

Due to an excess of acquisition artifacts, backscatter and curvature grids were discarded during terrain modelling. Grid size of model input data sets was set to 4 m since ROV video footage showed that substrate and associated communities vary at a very small scale, so larger grid sizes would fail at distinguishing small scale heterogeneity. Based on the general bathymetry of La Fonera canyon head, BPIs were calculated at two scales: (1) a fine-scale bathymetric position index raster (FS BPI) was computed for an inner radius of 4 m and an outer radius of 20 m (Fig 3C), and (2) a broad-scale bathymetric position index raster (BS BPI) was built with an inner radius of 8 m and an outer radius of 60 m (Fig 3D). Since the range of BPI values decreases with size of analysis window, BPI rasters were normalized by subtracting the mean value and dividing by the standard deviation. Benthic terrain modelling was then applied to 99.65 km^2^ of the bathymetric data set following an *ad hoc* classification schema (cf. section 4.1).

### ROV data

ROV video images were collected using a Seaeye Lynx 1500 during the months of November 2009, and May and June 2010, operated from *MV Bon Pigall* owned by *Fundació Argomaris*, designed for a depth range of 400 m. According to national regulations, no specific permissions were required for the use of ROVs in La Fonera canyon at the time of the study. ROV tracks were planned based on the bathymetric data aiming at obtaining enough information of all potential habitats in the canyon head. For each morphologically similar potential habitat, different locations were explored so that surveyed points are widely distributed within the canyon head. Safety was a basic criterion in such a rough terrain that is in addition littered with lost fishing gear posing a significant additional threat to the ROV [51]. *C*. *rubrum* is included in Annex V of Council Directive 92/43/EEC of 21 May 1992 on the conservation of natural habitats and of wild fauna and flora. No samples were collected of any species.

A total of 21 ROV immersions were carried out in La Fonera canyon head (Table 1). ROV tracking was achieved using a Seaquest Tracklink 1505 USBL acoustic tracking system integrated with the ship’s DGPS and motion sensor unit. During three of immersions, ROV positioning was lost due to system malfunctioning. Thus, 18 positioned ROV transects (Fig 2) were used to generate predictive maps of benthic habitats. The ROV was equipped with three video cameras: a low-resolution colour camera, a black and white long-range camera and a high-resolution colour camera. Images obtained by the first two were available on board live during the operations; the latter was only recovered when the ROV was back on board. A total of 420 Gb of video images were recorded (41 h 20 min approximately). Transects were performed upslope (i.e. from the canyon axis up to the canyon rim), at depths ranging between 79 and 401 m.

**Table 1:** Details of the ROV transects carried out in La Fonera canyon head.

Transect	Date	Time length	Length (km)	Depth range (m)	Target and comments
0N	240610	3:10	1.56	79-329	Illa Negra canyon floor
0S	240610	1:50	0.17	303-315	Illa Negra canyon floor
1N	250610	2:10	1.15	172-331	Cap de Begur canyon floor
1S	130610	0:30	0.20	300-394	Cap de Begur canyon floor. No colour HD recording
5	220510	1:10	-	-	Illa Negra floor, bad positioning
6	290510	2:40	0.28	126-348	North Illa Negra canyon wall
7	290510	1:20	0.51	98-304	North Illa Negra canyon wall
9	250610	1:50	0.39	125-392	South Illa Negra canyon wall
11	300510	1:40	0.72	96-313	South Illa Negra canyon wall
13	120610	0:50	0.25	90-189	North Illa Negra canyon wall
16	050610	2:50	0.94	110-299	West Cap de Begur canyon wall
17	050610	1:20	0.36	155-285	East Cap de Begur canyon wall
20	250610	1:40	0.58	190-393	East Cap de Begur canyon wall
22	300510	2:20	0.39	123-387	West Cap de Begur canyon wall
26	120610	1:20	-	-	North Sant Sebastià canyon wall, bad positioning
27	120610	1:20	0.42	106-306	South Sant Sebastià canyon wall
45	260610	2:30	0.60	163-366	North La Fonera canyon wall
47	151109	3:40	0.72	181-400	North La Fonera canyon wall
48	130610	2:50	1.02	184-371	North La Fonera canyon wall
49	120610	2:10	-	-	North La Fonera canyon wall, bad positioning
58	161109	2:10	0.83	207-338	North La Fonera canyon wall

Transects 5, 26 and 49 were not used to generate predictive benthic habitat maps since they suffered of ROV positioning problems.

ROV navigation was incorporated to an ArcGIS project together with bathymetric and backscatter data. After filtering position fixes where ROV was not in contact with the seafloor, 7914 position fixes depict the ROV track, that is, a mean of more than three fixes per minute of total recording time (Fig 2). Each second of video footage was classified in terms of substratum (mud or fine sediment, sand and rock), presence or absence of CWC, anthropogenic impacts (litter, litter type, trawling marks) and any other relevant observation. That means that a given position fix, spanning for some seconds, can contain multiple observations. In this study, “litter” encompasses all man-made objects such as ropes, longlines, nets and other fishing gear, plastic bags, bottles, cans, boxes, and also cultural heritage objects such as a Roman amphora.

With the aim to evaluate the correlation between cold-water and red coral observations and the bathymetric parameters (depth, gradient, rugosity, curvature, BS and FS bathymetric position index) as well as backscatter data, each of these parameters has been extracted for all position fixes with positive ROV observations.

### Maximum entropy prediction

Maximum entropy prediction of habitat suitability was performed with Maxent software, version 3.3.3k available online [53, 54], using the bathymetric parameters in combination with ROV positive observations of the three coral species considered in this study, namely *M*. *oculata*, *D*. *cornigera* and *C*. *rubrum*. In addition, the same model was used to predict litter accumulations using litter positive ROV observations. The parameters used were depth, gradient, rugosity, curvature, and fine scale and broad scale bathymetric position indexes. Backscatter data were not incorporated into the calculations since nadir noise and gain changes are too dominant for using data for quantitative analysis and obtaining proper predictions.

## Results

### Benthic terrain modelling

**Fig 4:**
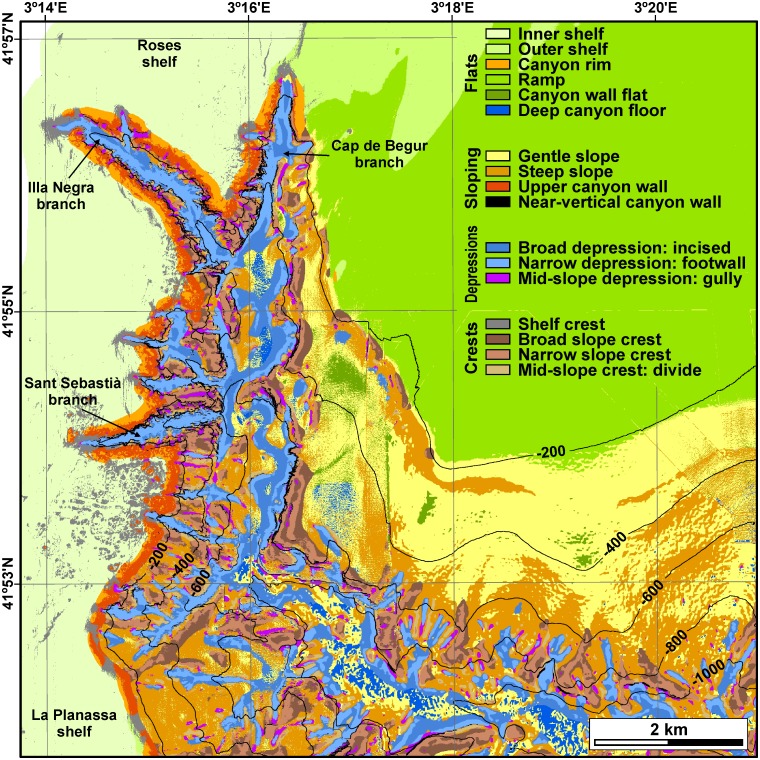
Benthic terrain classification of the study area. Note that the upper wall is well defined in the western canyon wall, and that near-vertical walls are located mostly at the lower parts of canyon walls.

The region over which the benthic terrain modelling has been applied comprises the La Fonera canyon head and surrounding shelf and slope (Fig 2). The canyon head down to an axial depth of 1000 m occupies mostly the western and southern parts, whereas a wide and smooth sloping shelf with a poorly defined shelf break extends over the northeastern part. Based on depth, slope and the two standardized BPI rasters, a classification scheme incorporating both margin-scale and fine structure elements has been specially designed for the study area (Table 2). When applied (Fig 4), it is able to differentiate between (1) flat areas on the shelf, walls and canyon floor, (2) sloping areas, (3) elevated areas on the shelf, walls (divides) and canyon floor, and (4) depressed areas on the canyon walls (gullies) or along the canyon axis.

**Table 2:** Classification scheme of the benthic terrain modelling of La Fonera canyon head and surrounding shelf and slope.

	Env	BS BPI (%)		FS BPI (%)		Gradient (°)		Depth (m)	
		LB	UB	LB	UB	LB	UB	LB	UB
**Flat areas**									
Inner shelf	Sh	-100	60	-100	60	0	6	-130	
Outer shelf	Sh	-100	60	-100	60	0	6	-160	-130
Canyon rim	Sh	60		-100		0	8	-160	
Ramp	Sl	-100	100	-100	100	0	6	-275	-160
Canyon wall flat	Ca	-100	100	-100	100	0	6	-475	-275
Canyon floor	Ca	-100	100	-100	100	0	6		-475
**Sloping areas**									
Upper wall #1	Ca	60		-100		8	60	-160	
Upper wall #2	Ca	-100	60	-100	60	20	60	-160	-120
Gentle slope	Ca	-100	100	-100	100	6	15		-160
Steep slope	Ca	-100	100	-100	100	15	60		-160
Near-vertical wall	Ca					60			
**Depressions**									
Broad depression: incised canyon	Ca		-100	-100	100	0	60		
Narrow depression: footwall	Ca		-100		-100	0	60		
Mid-slope depression: gully	Ca	-100			-100	0	60		
**Crests**									
Shelf crest #1	Sh	-100	60	-100	60	6	20	-160	
Shelf crest #2	Sh	-100	60	-100	60	20	60	-120	
Shelf crest #3	Sh		59	60		0	60	-160	
Broad slope crest	Ca	100		-100	100	0	60		-160
Narrow slope crest	Ca	100		100		0	60		-160
Mid-slope crest: divide	Ca		99	100		0	60		-160

Note a general classification (flat areas, sloping areas, depressions and crests) and how terrain classes are characterised within them based on specificities such as slope gradient (for slope) or depth (for flat areas). No overlapping occurs between classes. BS BPI: broad-scale bathymetric position index; Ca: canyon; Env: environment; FS BPI: fine-scale; LB: lower boundary; Sh: shelf; Sl: slope; UB: upper boundary.

Being overall a topographically rough area, flat areas are defined as those less than 6° in gradient (8° when including shallow depths, i.e. the canyon rim) and with BPI values within the first standard deviation (between -100 and 100), except for the shelf, where BPI values of 60 limit flats and shelf crests, and for the canyon rim, with positive BS BPI values. Flats are classified primarily based on their depth position. The 130 m isobath defines the limit between the inner and the outer shelf, and the 160 m isobath limits the outer shelf and the ramp, that is, a very gently sloping area connecting the Roses shelf and the upper open slope. All flats between 275 m and 475 m water depth are then considered canyon wall flats, and those located at depths exceeding 475 m represent flat-bottomed sections of the canyon floor.

Sloping areas are defined to exceed 6° in gradient (8° in shallow depths, i.e. the upper canyon wall) and to have BPI values also within one standard deviation, except for the upper canyon wall that, as it happens with the canyon rim, has positive BPI values due to their close location to the much deeper lower wall and canyon axis. Sloping areas are classified taking into account their gradient, characterizing gentle slopes below 15° and steep slopes above 15° and below 60°. Near-vertical walls are those cells with gradients exceeding 60°, disregarding depth and BPI values.

Depressed areas are accounted at all depth ranges and at all slope gradient ranges, except for near-vertical walls, when either one or both BPI’s are negative below one standard deviation. If BS BPI is below one standard deviation but FS is not, it is considered that the cell is located nearby the axis of an incised, v-shaped section of the canyon. Conversely, if BS BPI is within one standard deviation and FS is not, it can be assumed that the cell is in a local depression within a broader flat or homogeneously sloping area, for example a gully in the canyon wall. If both BPIs are negative below one standard deviation, the cell is located in a very narrow depression or at the foot of the canyon wall.

Crests are differentiated in shelf crests, located at depths shallower than 160 m and with BPI’s exceeding 60, and as slope crests and divides, at larger depths, when either one or both BPI’s are positive above one standard deviation. If FS, BS or both BPIs are positive, broad crests, narrow crests and crests within the canyon wall, that is, divides between gullies, are distinguished, respectively.

This semi-automatic terrain classification accurately describes the distribution of the different benthic terrains in the study area, with the exception of the flat canyon floor, seldom classified as gentle slope, and of the head tips of canyon branches, classified as shelf crests, which could be better described as shelf irregularities, disregarding their nature as either crests or depressions. The areal distribution of the different classes is indicated in Table 3. Within the study area, the ramp is the most common terrain class (29.1% of all cells), extending over the very gentle sloping area connecting the Roses shelf and the upper open slope (Fig 4), followed by the flat inner shelf (16.4%), representing to the smooth and sedimented regions of the inner shelf, without accounting shelf crests and irregularities, which barely sum 2%. Almost 51.7% of the cells correspond to flat areas and almost 28.6% to sloping areas, among which 1% (0.94 km^2^) have gradients exceeding 60°. Depressions account for 10.1% of the classified area, but it is worth noting that some parts of the canyon axis are classified as flat canyon floors. Crests represent 9.6% of the area. Flat and uniformly sloping regions such as the shelf or a regular slope (e.g., the ramp and the eastern wall) are also characterised by curvature values close to zero (Fig 3E), whereas those places where the seafloor gradient abruptly changes have high positive or negative curvature values, denoting terrain irregularities such as complex, gullied canyon walls or escarpments. Rugosity values close to 1 (Fig 3F) are indicative of zones whose 3D area equals their plan area, and thus rather simple, homogeneous topographies; on the contrary, high rugosity values over 10 indicate regions whose 3D area is ten times the size of their plan area (e.g. canyon walls of Illa Negra and Sant Sebastià branches), providing an idea of a highly complex and heterogeneous seascape. The three bathymetric-derived data sets (curvature, rugosity and benthic terrain classification) provide a complete picture of the terrains in La Fonera canyon, their distribution and heterogeneity.

**Table 3:** Areal distribution of the different terrain classes identified in La Fonera canyon head and nearby shelf and slope.

	Cell number	Area (km^2^)	Area (%)	ROV observations (%)
**Flat areas**				
Inner shelf	1,020,561	16,328,976	16.4	9.8
Outer shelf	202,466	3,239,456	3.3	0
Canyon rim	66,360	1,061,760	1.1	6.7
Ramp	1,812,287	28,996,592	29.1	0.6
Canyon wall flat	40,990	655,840	0.7	0.1
Canyon floor	75,875	1,214,000	1.2	0
**Sloping areas**				
Upper wall (aggregated)	106,136	1,698,176	1.7	17.6
Gentle slope	810,347	12,965,552	13.0	14.6
Steep slope	807,911	12,926,576	13.0	13.3
Near-vertical wall	58,624	937,984	0.9	2.3
**Depressions**				
Broad depression: incised canyon	257,848	4,125,568	4.1	12.1
Narrow depression: footwall	277,921	4,446,736	4.5	9.1
Mid-slope depression: gully	91,556	1,464,896	1.5	0.5
**Crests**				
Shelf crest (aggregated)	100,645	1,610,320	1.6	2.7
Broad slope crest	154,735	2,475,760	2.5	3.7
Narrow slope crest	226,140	3,618,240	3.6	6.0
Mid-slope crest: divide	117,603	1,881,648	1.9	0.9
Unclassified	74	1184	0.0	0

Unclassified cells are those that their specific values (e.g. exact depth of 130 m) make them attributable to two or more classes (e.g. inner and outer shelf).

ROV inspection effort was focused on specific terrain classes within the study region (Table 4). Inspection efforts concentrated on the canyon rim (6.7% of position fixes with respect to 1.1% of terrain class extension), upper wall (17.6% of position fixes with respect to 1.7% of terrain class extension) and the floor of the incised branches (12.1% of position fixes with respect to 4.1% of terrain class extension). Near-vertical walls were also inspected (2.3% of position fixes) since they were good candidate locations for CWC. On the contrary, very few images were obtained from the ramp (0.6%) even though it is the most common terrain class in the study area, and no ROV images were obtained from the outer shelf. The main reason is that taking into account background information from nearby canyons (cf. section 2), these two terrain classes have very little chances to host significant CWC colonies. Also, the deep canyon floor remained uninspected as it was beyond the ROV depth range.

**Table 4:** ROV inspection effort for each terrain class along each transect and for the whole survey.

Dive	PF	PF	Inner shelf	Canyon rim	Ramp	Canyon wall flat	Upper wall	Gentle slope	Steep slope	Near-vertical wall	Incised canyon	Footwall	Gully	Shelf crest	Broad slope crest	Narrow slope crest	Divide
	(N)	(%)	(%)	(%)	(%)	(%)	(%)	(%)	(%)	(%)	(%)	(%)	(%)	(%)	(%)	(%)	(%)
0N	918	11.6	-	-	-	-	-	-	0.4	0.3	62.6	35.7	0.1	0.8	-	-	-
0S	29	0.4	-	-	-	-	-	-	-	-	6.9	93.1	-	-	-	-	-
1N	243	3.1	-	-	-	-	-	-	0.8	-	53.5	28.8	-	-	-	16.1	0.8
1S	83	1.0	-	-	-	-	-	-	-	3.6	-	95.2	-	-	-	1.2	-
6	439	5.5	-	-	-	-	41.0	-	5.7	2.5	8.7	9.1	0.5	-	1.4	26.9	4.3
7	948	12.0	7.6	33.9	-	-	27.1	0.1	17.2	0.4	12.8	-	0.6	-	0.3	-	-
9	458	5.8	-	8.3	-	-	50.9	-	1.5	8.7	-	1.1	1.7	-	0.2	23.4	4.1
11	506	6.4	56.3	5.3	-	-	30.6	-	0.2	-	0.6	6.1	-	-	0.6	-	0.2
13	143	1.8	-	7.7	-	-	39.9	-	1.4	4.2	40.6	-	4.2	0.7	-	-	1.4
16	499	6.3	67.0	4.8	-	-	7.8	-	-	0.4	0.6	0.4	0.8	13.6	0.8	0.6	0.2
17	184	2.3	-	28.8	-	-	12.5	-	-	7.6	5.4	10.9	3.8	-	-	29.9	1.1
20	107	1.4	-	-	-	-	-	14.0	17.8	0.9	-	13.1	-	-	15.0	30.8	8.4
22	273	3.4	-	5.1	-	-	75.5	-	-	8.8	-	2.2	-	-	0.4	6.6	1.5
27	698	8.8	10.0	6.0	-	-	34.8	-	0.7	9.7	2.4	14.5	-	19.3	-	1.3	1.1
45	1250	15.8	-	-	0.1	-	-	41.3	35.8	0.2	-	-	0.1	-	17.6	5.0	-
47	140	1.8	-	-	22.9	-	-	20.0	30.0	-	-	-	-	-	24.3	2.1	0.7
48	817	10.3	-	-	2.0	1.1	-	51.8	40.6	0.7	-	-	0.1	-	0.1	3.1	0.5
58	179	2.3	-	-	0.6	1.1	-	96.1	2.2	-	-	-	-	-	-	-	-
Total (N)	7914	-	776	530	50	11	1393	1155	1053	185	957	723	36	211	289	473	72
Total (%)	-	100	9.8	6.7	0.6	0.1	17.6	14.6	13.3	2.3	12.1	9.1	0.5	2.7	3.7	6.0	0.9

Terrain classes “outer shelf” and “canyon floor” were not inspected because of the low chances of occurrence of CWC and their position beyond the depth range of the ROV, respectively. For the same reasons, inspection effort in other classes (e.g. ramp, canyon wall flat) was limited. PF: position fixes

### Substrate types

**Fig 5:**
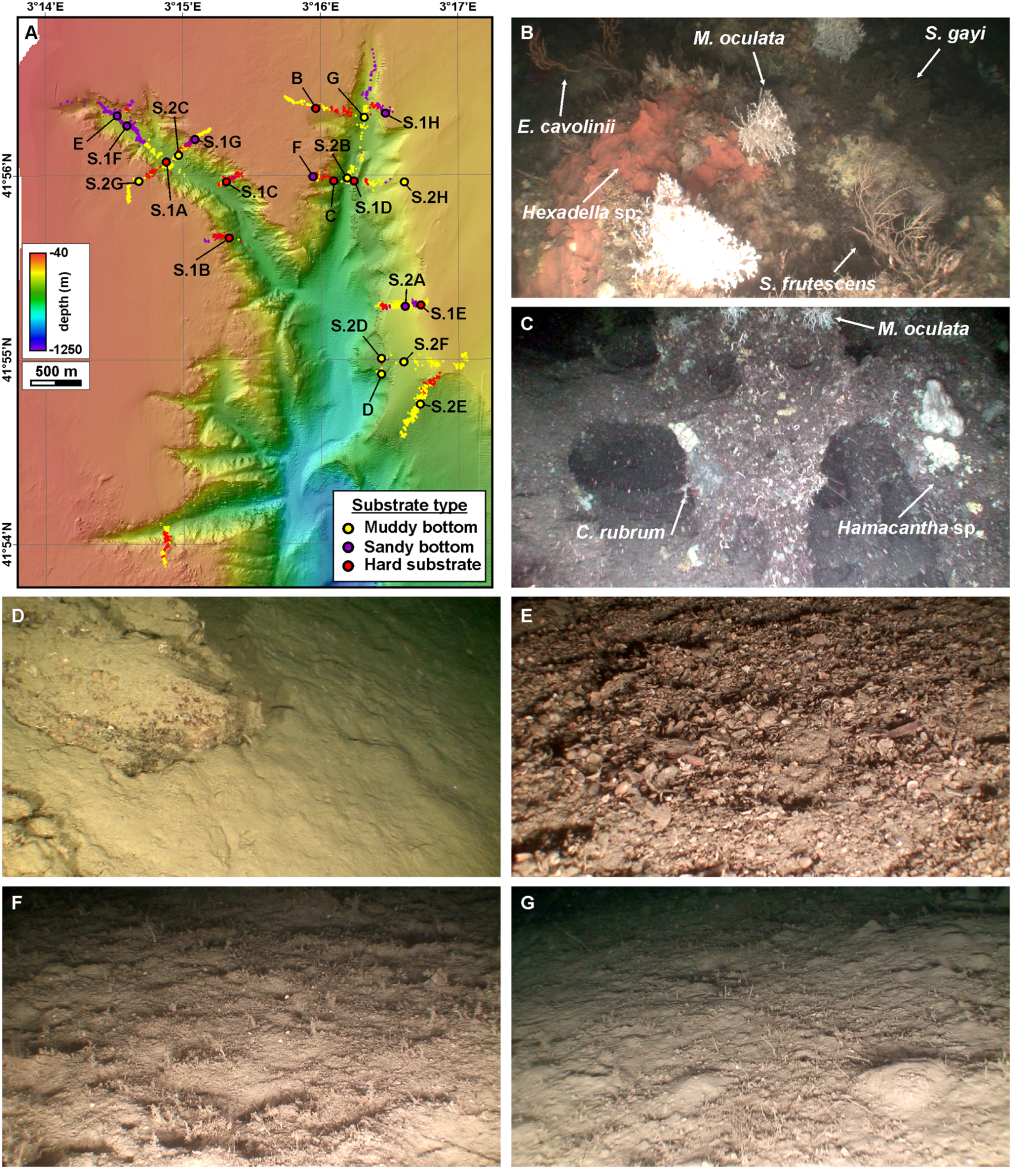
Substrate types in the study area. A: Substrate types observed along ROV dives: muddy bottom, sandy bottom and hard substrate. See text for clarification of this classification. Locations of ROV images in this figure (B to G) as well as in S1 and S2 Figs are provided. B: Rock outcrops on the western wall of Cap de Begur branch (134 mwd, upper wall) with a variety of organisms. C: Rock outcrops with overhangs and caves on the western wall of Cap de Begur branch (290 mwd, near-vertical wall), with small *M*. *oculata* and *C*. *rubrum* colonies. D: Rock blocks covered by a cm-thick mud veneer on a sedimented area at the eastern wall of the canyon close to Sant Sebastià fishing ground (391 mwd, divide). E: Bioclastic sand and fine gravel along the axis of Illa Negra branch (182 mwd, incised canyon). F: Sandy bottom in the western rim of Cap de Begur branch (123 mwd, canyon rim) with polychaete *Lanice conchilega*. G: Muddy bottom with some *L*. *conchilega* specimens along the axis of Cap de Begur branch (297 mwd, incised canyon).

ROV images display a notable habitat variety, in terms of bottom topography and substrate types, in La Fonera canyon head and branches. ROV observations have allowed classifying the substrate of the study area into three broad groups: (1) rocky outcrops and large boulders, (2) sandy bottoms and (3) fine sediment-covered bottoms (Fig 5). Given the limitations of this classification, especially regarding the distinction of grain sizes, the second and third categories could be more accurately described and as coarse sand to fine gravel and as clay to medium sand mixtures bottoms, respectively. For ease of simplicity and conciseness we will refer to “hard substrate”, “sandy bottom” and “muddy bottom” from here onwards.

Hard substrates are represented either by rocky outcrops or large boulders. Rocky outcrops dominate the lower and middle walls in the western canyon branches and gullies, as well as the eastern wall of Cap de Begur branch. They also form some linear escarpments along the eastern La Fonera canyon wall at the western end of Sant Sebastià fishing ground (Fig 5A). Hard substrate communities are formed by a variety of encrusting organisms (Fig 5B). Very irregular rock formations, with small caves and holes occupy the lower and middle part of the western wall of Cap de Begur branch, often forming overhangs (Fig 5C), whereas more regular, sub-rounded morphologies are imaged along Illa Negra branch (S1A and S1B Fig). The upper parts of the walls are more often occupied by decimetric to metric boulders, frequently with a thin mud cover (S1C Fig), which attains a relevant thickness able to smooth the original rocky relief in the linear escarpments near the Sant Sebastià fishing ground (Fig 5D). The transition between rock outcrops and muddy bottom is abrupt at the foot of the walls especially in the inner parts of the canyon head (S1D Fig), although substrate changes are generally gradual (S1E Fig).

Sandy bottoms are mainly found in two environments: at the canyon floor along the axes of Illa Negra and Cap de Begur branches at distances shorter than 1 km from their tips and depths below 260 m, and on the canyon rims around these two branches (Fig 5A). The first one is characterised by coarse bioclastic sand and gravel (Figs 5E and S1F), whereas sand is finer on the second environment (Figs 5F, S1G and S1H). Bioclastic sandy bottoms can also be found associated to the linear escarpments along the eastern La Fonera canyon wall (S2A Fig).

Muddy bottoms extend within the canyon along the axes of the two main branches at depths exceeding 260 m (Figs 5A, 5G, S2B and S2C) and on the smooth areas of the eastern canyon wall (S2D, S2E and S2F Fig). Outside the canyon, muddy bottoms occupy the inner shelf areas away from the canyon rim (Figs 5A and S2G) and the upper slope ramp east of the canyon head (S2F Fig).

Most ROV observations were made over muddy bottoms (54%), while sandy bottoms and hard substrates were surveyed equally (22% and 24%, respectively) (Table 5). Regarding the terrain classes defined on the basis of benthic terrain modelling, muddy bottoms cover all (100%) the surveyed inner shelf, ramp and canyon wall flats, and most (>80%) of the shelf crests, gentle slopes and broad slope crests. Muddy bottoms are also the dominant bottom type (>45%) in the canyon rims, footwalls and steep slopes, although a significant number of observations (>12%) in these terrain classes also display sandy bottoms. Sandy bottoms occupy most (67%) of the incised canyon terrain class, although a significant part is covered by mud (26%). Hard substrates characterize most of near-vertical walls (84%), and a majority (>50%) of upper walls, gullies, divides and narrow slope crests (Table 5).

**Table 5:** Substrate types, CWC, red coral and litter observations for each terrain class.

ROV observations	PF (N)	PF (%)	Inner shelf	Canyon rim	Ramp	Canyon wall flat	Upper wall	Gentle slope	Steep slope	Near-vertical wall	Incised canyon	Footwall	Gully	Shelf crest	Broad slope crest	Narrow slope crest	Divide
Total (N)	7914		776	530	50	11	1393	1155	1053	185	957	723	36	211	289	473	72
Substrate type			(%)	(%)	(%)	(%)	(%)	(%)	(%)	(%)	(%)	(%)	(%)	(%)	(%)	(%)	(%)
Muddy bottom	4276	54.03	100	64.5	100	100	19.1	97.4	45.5	15.1	26.4	55.6	25.0	88.6	84.8	19.4	15.3
Sandy bottom	1740	21.99	0	23.0	0	0	16.1	1.6	34.3	1.1	67.0	31.7	22.2	10.9	6.9	18.0	8.3
Hard substrate	1898	23.98	0	12.5	0	0	64.8	1.0	20.2	83.8	6.6	12.7	52.8	0.5	8.3	62.6	76.4
CWC and red corals			(%)	(%)	(%)	(%)	(%)	(%)	(%)	(%)	(%)	(%)	(%)	(%)	(%)	(%)	(%)
*M*. *oculata*	270	3.41	0	0.4	0	0	2.2	0.5	4.9	29.2	0.9	4.8	36.1	0	2.4	11.8	6.9
*D*. *cornigera*	121	1.53	0	0.6	0	0	1.7	0.4	4.7	2.2	0.7	0.6	11.1	0	1.7	2.7	2.8
*C*. *rubrum*	169	2.13	0	0.8	0	0	7.8	0.9	0.9	11.4	0	0.1	0	0.5	0	5.1	0
Litter	604	7.63	3.7	2.3	0	0	17.5	0.9	7.8	17.3	0.9	5.3	2.8	15.2	4.8	19.7	11.1

Values are shown in percentages over the number of observations for each terrain class, indicated in the first row. See text for clarification of substrate types. PF: position fixes.

### CWC observations

The dominant CWC species in La Fonera canyon head is *M*. *oculata*, which at some locations forms dense middle-sized colony clusters, often accompanied by several other species of megafauna that are easily identifiable with the ROV images. Gorgonians *Eunicella cavolinii* and *C*. *rubrum*, and corals *D*. *cornigera* and *Caryophyllia smithii* are usually associated to *M*. *oculata*. Other sessile species growing over the hard substrates or coral skeletons include several species of sponges (*Poecillastra compressa*, *Hexadella* spp., *Hamacantha* spp., *Hymedesmia* sp., *Crella* spp., *Reniera* spp., *Axinella damicornis*), hydrozoans (*Sertularella gayi* and *Schizotricha frustescens*), bryozoans (*Reteporella* sp.), polychaetes (*Salmacina dysteri*, *Sabella pavonina*, *Vermiliopsis* sp. and *Protula tubularia*), brachipods (*Gryphus vitreus*) and the bivalve *Neopycnodonte cochlear* forming dense clusters. We have also observed several vagile species roaming over or between the corals such as spiny lobsters (*Palinurus elephas*), squad lobsters (*Munida* sp.), hermit crabs (*Dardanus arrosor*), sea urchins (*Echinus* spp., *Cidaris cidaris*), sea cucumbers (*Holothuria forskali*) and octopus (*Octopus salutii*). Basket star *Astropartus mediterraneus* and dense populations of ophiurioid *Ophiothrix* sp. have been observed attached to coral branches.

**Fig 6:**
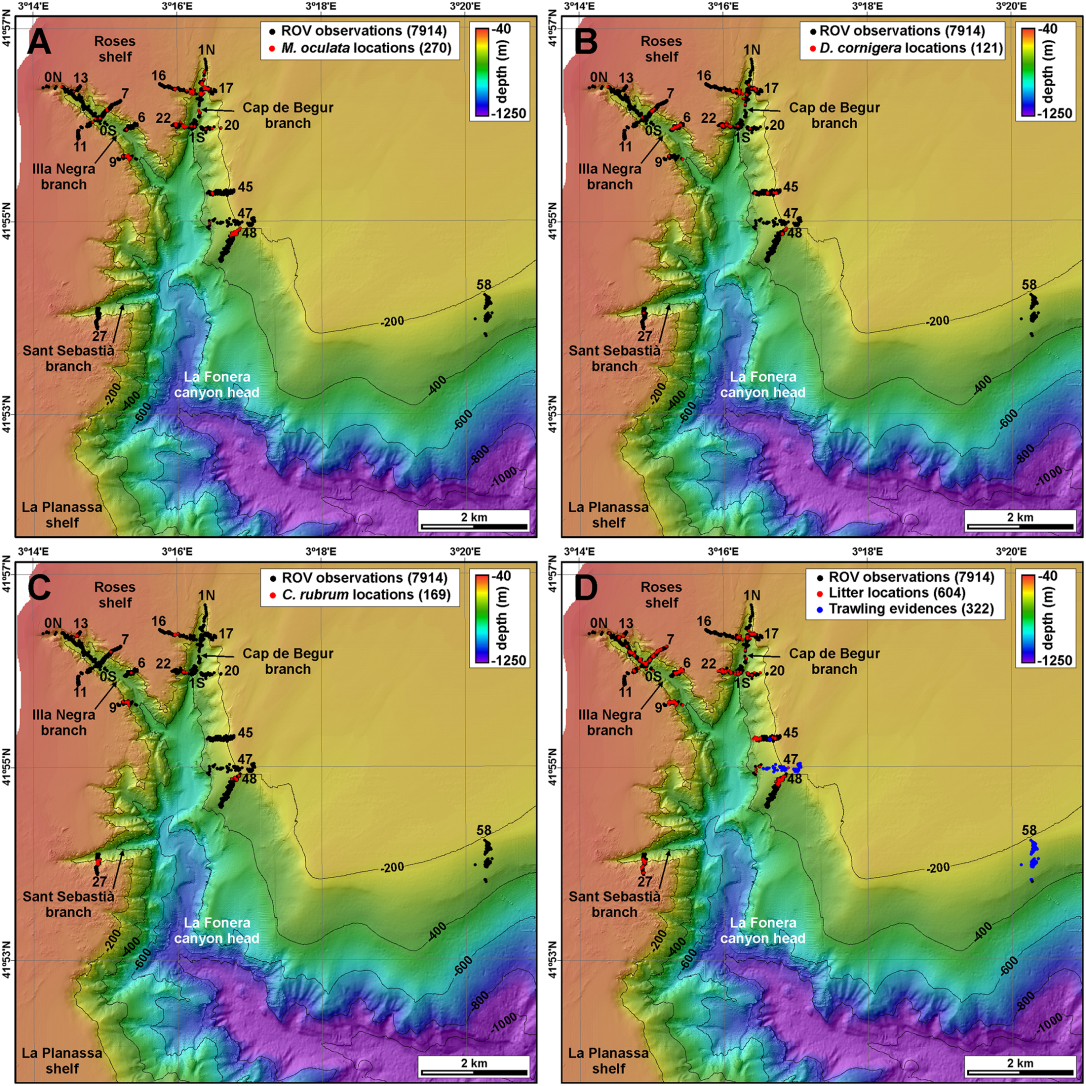
ROV observations of corals and human impacts. A: Positive observations of living *M*. *oculata* colonies. B: Positive observations of living *D*. *cornigera* colonies. C: Positive observations of living *C*. *rubrum* colonies. D: Positive observations of litter (all types) and bottom trawling evidences on the seafloor.

*M*. *oculata* was observed in 12 out of the 21 ROV immersions, *D*. *cornigera* in 14 and *C*. *rubrum* in 10 of them. Out of a total of 7914 position fixes, *M*. *oculata* colonies have been imaged in 270 of them (3.41%), *D*. *cornigera* in 121 (1.53%) and *C*. *rubrum* in 169 (2.13%), but they are far from being homogeneously distributed within the canyon environments (Fig 6). Observations do not account for colony health, i.e., a positive observation could indicate the presence of a single, small, isolated, broken or sediment capped colony, whereas another positive observation could refer to a well-developed, large colony.

**Fig 7:**
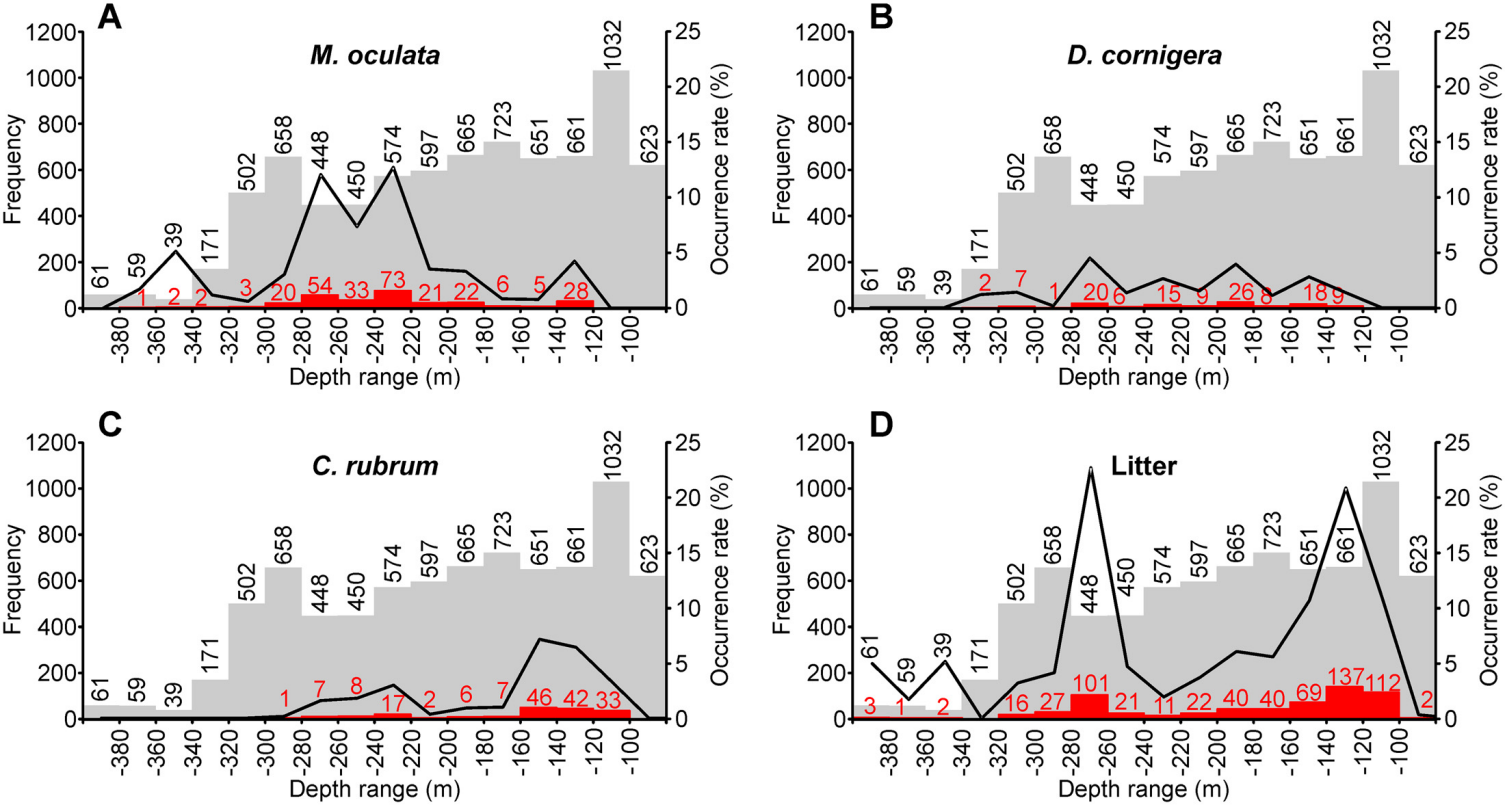
Frequency and occurrence rate vs. depth of observed corals and litter. A: *M*. *oculata* by depth range. B: *D*. *cornigera* by depth range. C: *C*. *rubrum* by depth range. D: Litter items by depth range. In all panels, background grey histogram indicates frequency of ROV observations, whereas red histogram indicates frequency of observation of each species or litter items. Black line indicates occurrence rate, that is, number of positives with respect to total observations for each depth range.

**Fig 8:**
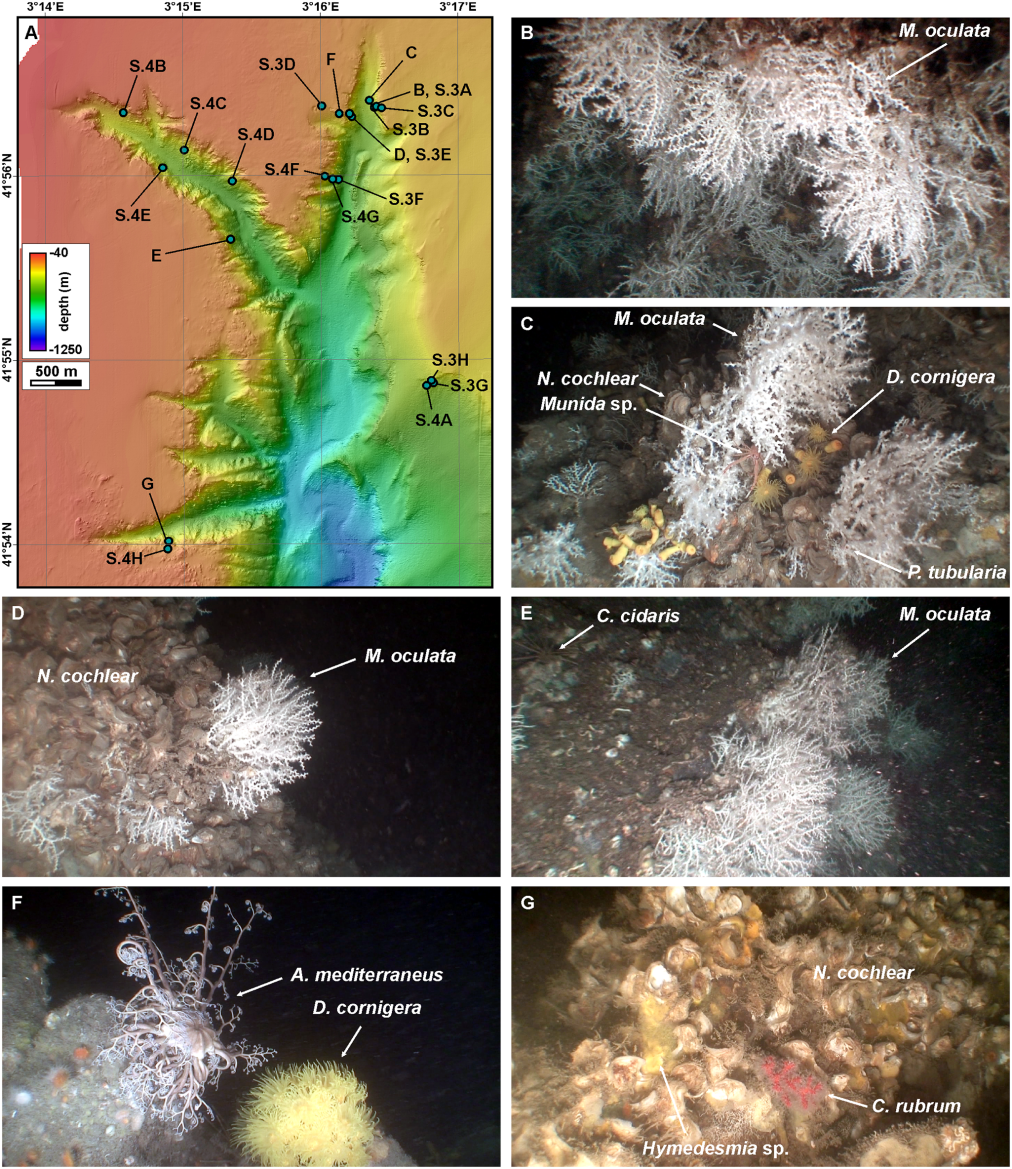
Cold-water and red coral colonies imaged by ROV. A: Locations of ROV images in this figure (B to G) as well as in S3 and S4 Figs. B: Dense overhanging *M*. *oculata* living colonies at the foot of Cap de Begur eastern wall (205 m, near-vertical wall). C: Dense *M*. *oculata* and *D*. *cornigera* living colonies at the foot of Cap de Begur eastern wall, with decapod *Munida* sp., polychaete *P*. *tubularia* and deep-water oyster *N*. *cochlear* (267 m, footwall). D: *M*. *oculata* colonies over an oyster-covered rock (*N*. *cochlear*) on the western wall of Cap de Begur branch (223 m, near-vertical wall). E: Dense *M*. *oculata* living colonies on the western wall of Illa Negra branch, with echinoderm *C*. *cidaris* (230 m, steep slope). F: *D*. *cornigera* living colony next to echinoderm *A*. *mediterraneus* on the upper part of the western wall of Cap de Begur branch (132 m, upper wall). G: *C*. *rubrum* living colony on a *N*. *cochlear*-covered rock on the southern wall of Sant Sebastià branch (141 m, upper wall).

*M*. *oculata* colonies span over water depths ranging between 129 and 369 m (mean 230 m), and are most frequent between 180 and 300 m water depth (Fig 7), on highly sloping areas (mean 42.8°). Observed *M*. *oculata* colonies form apparently fragile frameworks but are in general in good shape except when they are directly or indirectly impacted by human activities (cf. section 4.4), and preferentially develop on protected sections of the walls, often in overhangs (S1 Video). Thus, most sightings of these species were made in the most abrupt parts of the canyon walls, although some were made in places like the canyon axis. These locations are in general less exposed to impacts from commercial trawling. Medium-sized, apparently decimetric colonies develop on the eastern wall of Cap de Begur branch, being especially dense at the foot of the canyon wall where they look like hanging palm tree forests (Figs 8B and S3A). These colonies often co-occur with *D*. *cornigera* (Figs 8C and S3B) and host a wide variety of fauna including polychaete *P*. *tubularia*, ophiurioid *Ophiothrix* sp., oyster *N*. *cochlear*, echinoderm *C*. *cidaris*, decapods *Munida* sp. and *P*. *elephas*, holothurians and bony fishes (Figs 8C and S3C). *M*. *oculata* colonies also develop just opposite across the Cap de Begur branch on the western wall, although less densely (Fig 8D). In this wall, colonies reach shallower water depths (130 m, S3D Fig), also living together with Paguroidea and deep-water oysters *N*. *cochlear* (Figs 8D and S3E). *M*. *oculata* colonies extend further south on the western wall, as imaged during transect 22 (Figs 5C and S3F), than on the eastern wall, where they were no longer detected during transect 20 (Fig 6A). Nevertheless, beyond the confluence of the Cap de Begur and Illa Negra branches, *M*. *oculata* colonies appear back on the uppermost section of the eastern wall, very close to Sant Sebastià fishing ground (Fig 6A). There, they coexist with *D*. *cornigera* and *C*. *rubrum* colonies (S3G and S3H Fig) and even grow over lost fishing gear (S4A Fig). Colonies of *M*. *oculata* are much scarcer in Illa Negra branch, and have not been observed in Sant Sebastià branch (Fig 6A). In spite of their scarcity, medium-sized living colonies were found during transect 9 along the middle and upper sections of the western wall of Illa Negra branch, nearby the confluence with Cap de Begur branch (Figs 6A and 8E).

*D*. *cornigera* colonies are sparser but evenly distributed at depth, ranging between 131 and 325 m water depth (mean 211 m) (Fig 7), on relatively low sloping areas (mean 29.3°). Sometimes found in association with *M*. *oculata* colonies (Figs 8B, S3B, S3C and S3H), they are more often observed as isolated 5–10 cm large colonies of a few individuals (Fig 8F) or as a single polyp (S4B Fig). As such, it is relatively abundant along the walls of Illa Negra branch (Figs 6B and S4B–S4E), but also appears on Cap de Begur (Figs 6B and S4F) and Sant Sebastià branches, as well as on the uppermost section of the eastern La Fonera canyon wall (S1E Fig), generally on sediment-covered rocks along gently sloping areas.

*C*. *rubrum* colonies are slightly more frequent than those of *D*. *cornigera*. They appear between 101 and 297 m water depth (mean 156 m), but are most frequent at the shallower part of this interval, between 100 and 160 m (Fig 7), on highly sloping areas (mean 43.2°). Only at a very small number of locations polyps were observed, like in Fig 8G, where *C*. *rubrum* grows on a *N*. *cochlear* deep-water oyster bank. In general, they appear as small, centimetric colonies located in highly protected places, such as overhangs and caves (S4G and S4H Fig), which are far from reaching the size or the density of the well-developed *M*. *oculata* colonies. At 28% of locations where *C*. *rubrum* was found it was observed to coexist with *M*. *oculata* (S3G Fig), and only at two locations were the three species found together (S3H and S4B Figs).

Each positive observation has been related with the benthic terrain model classification. This allows identifying if each species concentrates on any of the benthic terrain classes. *M*. *oculata* colonies have been most reported on narrow slope crests (56 position fixes), near-vertical walls (54), steep slopes (52) and footwalls (35), although taking into account the number of observations carried out over each terrain class, this species preferentially settles on gullies (36% of observations on gullies included *M*. *oculata* specimens), near-vertical walls (29%) and narrow slope crests (12%) (Table 5). *D*. *cornigera* colonies have been most observed on steep slopes (50 position fixes) and upper canyon walls (24), and taking into account the number of observations carried out over each terrain class preferentially settle on gullies (11% of observations on gullies included *D*. *cornigera* specimens) and steep slopes (5%). Contrarily to *M*. *oculata*, *D*. *cornigera* colonies are seldom observed on near-vertical walls (Table 5). Finally, ROV images mostly show red corals on upper canyon walls (108 position fixes), narrow slope crests (24) and near-vertical walls (21), and taking into account the number of observations carried out over each terrain class they are more abundant on near-vertical walls (11% of observations on near-vertical walls included *C*. *rubrum* specimens) and upper canyon walls (8%) (Table 5). No specimens of these three species have been observed on the inner shelf, ramp, canyon wall flats, and only twice red corals have been observed on shelf crests.

Minimum, maximum and mean values of bathymetric parameters (depth, gradient, rugosity, curvature, BS and FS bathymetric position index) at locations were cold-water and red corals as well as litter have been observed are provided in Tables 6 and 7. Significant differences between the bathymetric locations and the characteristics of the terrains where these three species settle stand out. *M*. *oculata* colonies appear at a mean depth of 230 m, deeper than *D*. *cornigera* (211 m mean depth) and especially if compared to *C*. *rubrum* (156 m mean depth) (Table 6). Both *M*. *oculata* and *C*. *rubrum* colonies develop in high gradient slopes, with mean values of 42.8° and 43.2°, respectively, which are much higher than the mean slope gradient were *D*. *cornigera* establishes (29.3°). There is also a significant difference in the mean curvature of the terrain where *M*. *oculata* establishes, with a value of 9.4 compared to where *D*. *cornigera* develops (-4.1) and the mean of all points within the study area (0.2). Backscatter values are also more elevated in areas colonized by *M*. *oculata* and *D*. *cornigera* (Table 6). The presence of all coral species is also related to more positive values of both broad scale and fine scale BPIs (Table 7). The most significant differences are those for *C*. *rubrum* colonies, which develop in areas with mean FS BPI values (405.6) and mean BS BPI values (230.1), almost ten times above the general value. All species develop in areas where rugosity is well over the mean (1.80 for *M*. *oculata*, with respect to a mean value of 1.19 for the study area) (Table 7).

**Table 6:** Bathymetric parameters (depth, gradient, curvature) and backscatter of the grid cells.

Observations	N	Depth (m)				Gradient (°)				Curvature		Backscatter	
		Min	Max	Mean	SD	Min	Max	Mean	SD	Mean	SD	Mean	SD
Total	7914	-79	-401	-197	74.0	0.3	80.7	20.5	15.7	0.2	30.4	153	49.2
*M*. *oculata*	270	-129	-369	-230	47.8	4.4	80.7	42.8	19.2	9.4	76.8	162	62.2
*D*. *cornigera*	121	-131	-325	-211	52.1	4.3	80.7	29.3	14.1	-4.1	47.5	183	46.7
*C*. *rubrum*	169	-101	-297	-156	48.1	3.3	72.3	43.2	14.8	0.8	65.8	142	44.3
Litter	604	-99	-394	-183	67.1	0.8	73.6	27.6	16.6	1.1	33.8	155	45.8

“Total” refers to all cells, other rows refers to those cells with positive observations of cold-water and red corals and litter.

**Table 7:** Bathymetric parameters (rugosity, broad scale and fine scale bathymetric position index) of the grid cells.

Observations	N	Rugosity		BS BPI 240 8-60				FS BPI 80 4-20			
		Mean	SD	Min	Max	Mean	SD	Min	Max	Mean	SD
Total	7914	1.19	0.40	-631	416	21.3	202.3	-749	893	48.9	179.5
*M*. *oculata*	270	1.80	0.89	-456	388	36.5	177.6	-574	586	94.0	249.4
*D*. *cornigera*	121	1.34	0.68	-533	388	75.1	169.5	-749	521	86.4	180.7
*C*. *rubrum*	169	1.71	0.57	-324	388	230.1	122.3	-443	783	405.6	259.5
Litter	604	1.31	0.47	-512	409	162.9	208.2	-443	783	198.5	229.2

“Total” refers to all cells, other rows refers to those cells with positive observations of cold-water and red corals and litter.

### CWC prediction

**Fig 9:**
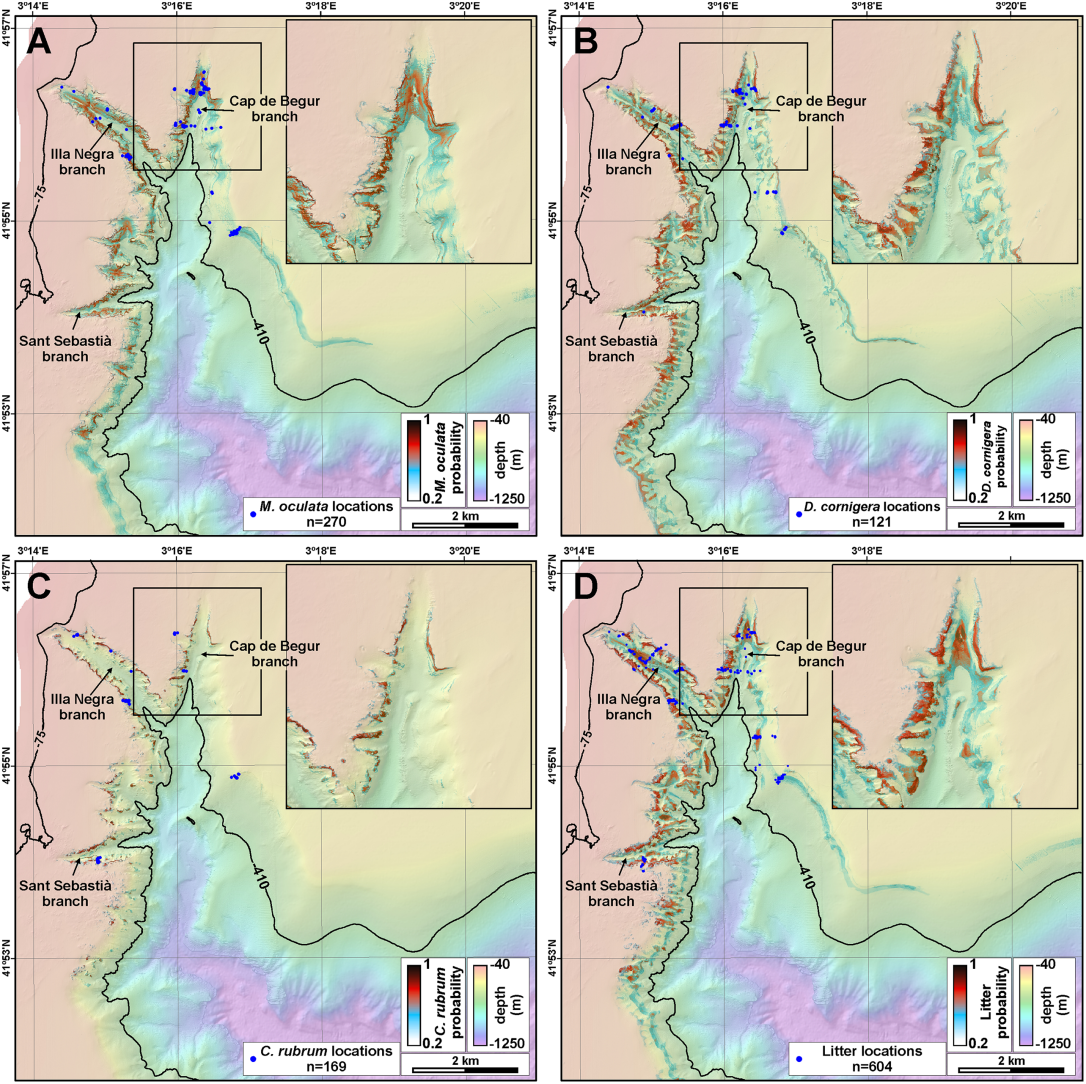
Maximum entropy prediction in La Fonera canyon head. A: *M*. *oculata* occurrence probability. B: *D*. *cornigera* occurrence probability. C: *C*. *rubrum* occurrence probability. D: Litter occurrence probability.

A predictive model of *M*. *oculata* occurrence in La Fonera canyon head by maximum entropy modelling is presented in Fig 9A. Modelled occurrence probability of this species is high on the highly sloping sections of Cap de Begur and Illa Negra branches. Some 0.36 km^2^ out of 6.94 km^2^ of the modelled area between 75 and 410 m water depth yield probabilities over 70%, and 0.10 km^2^ exceed 80% probability of occurrence of *M*. *oculata* colonies.

Similar predictive models have been produced for *D*. *cornigera* (Fig 9B) and *C*. *rubrum* (Fig 9C). Occurrence probability of the first is in general higher than *M*. *oculata*, and its spatial distribution is broader. A total of 0.43 km^2^ have probabilities over 70%, and 0.12 km^2^ exceed 80% probability of occurrence of *D*. *cornigera* colonies. Its presence is highly dependent on local morphological changes in the slope; in general, it is absent in depressions (Fig 9B). On the contrary, distribution of *C*. *rubrum* is much more restricted in space, with occurrence probabilities exceeding 70% extending over 0.15 km^2^, but occurrence probabilities have higher values (1904 m^2^ exceed 95%) at shallower depths than the other two species, especially on the upper parts of the canyon walls. The model predicts that this species is mostly restricted to the western wall of the canyon head, and should be almost absent in the eastern wall (Fig 9C). Jackknife tests of variable importance show that the variables that appear to have the most useful information by themselves regarding the presence of *M*. *oculata* and *D*. *cornigera* are seafloor gradient and rugosity, whereas for *C*. *rubrum*, these variables are fine-scale bathymetric position index and rugosity (S5 Fig). For the three species, the main environmental variable is water depth (S5 Fig).

### Anthropogenic impacts

**Fig 10:**
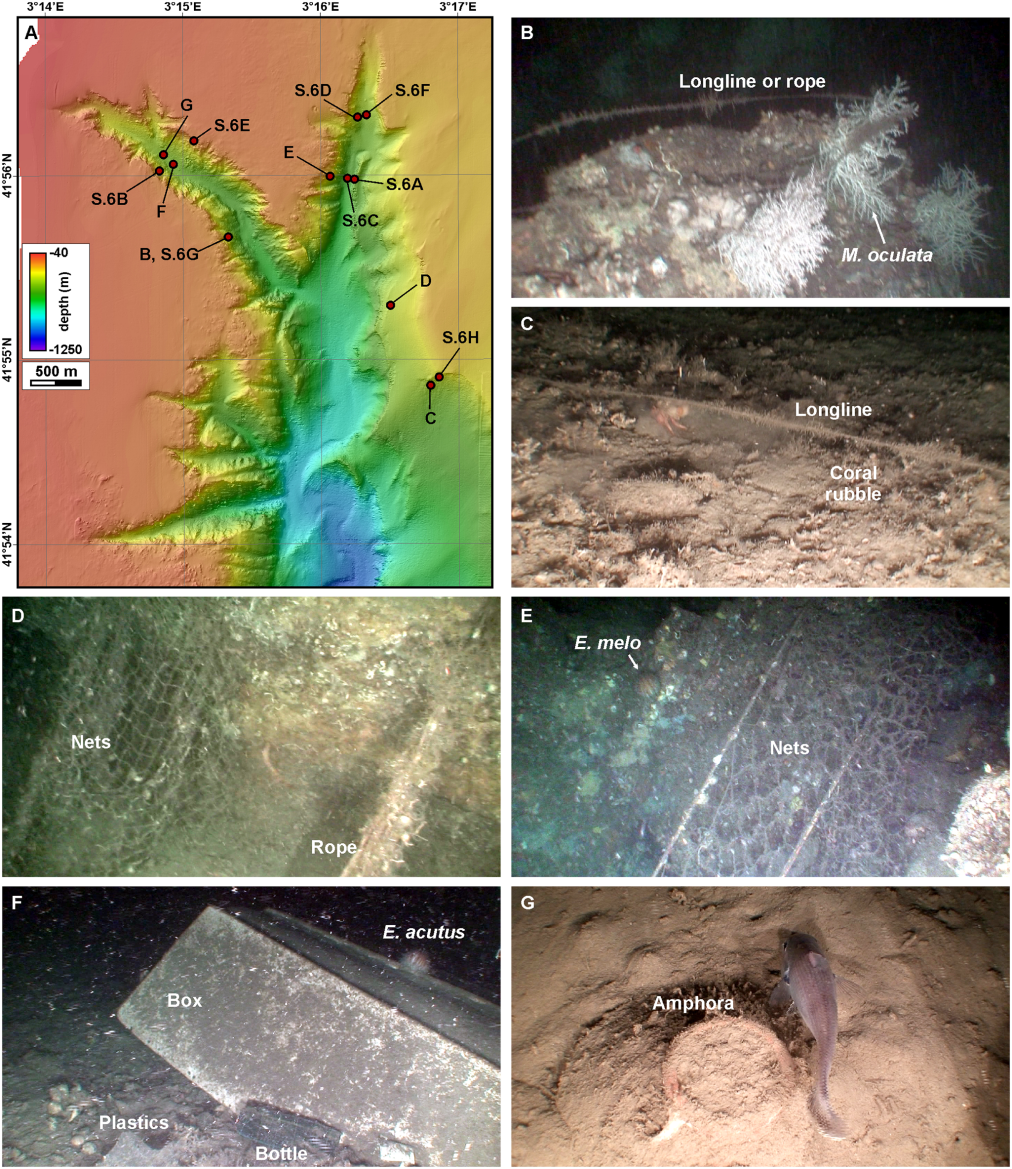
Examples of litter observed on the seafloor of the study area. A: Locations of ROV images in this figure (B to G) as well as in S6 Fig. B: Longline entangled on *M*. *oculata* colonies on the western wall of Illa Negra branch, next to the colonies imaged in Fig 8E (231 m, near-vertical wall). C: Longline resting over dead coral rubble at the foot of a coral colony (next to S3G, S3H and S4A Figs) on the upper part of the eastern La Fonera canyon head wall (273 m, steep slope). D: Fishing net entangled on rock on the eastern wall of La Fonera canyon (256 m, broad slope crest). E: Large fishing net entangled on the rocks outcropping on the western wall of Cap de Begur branch, with *Echinus melo* (207 m, near-vertical wall). F: Large plastic box, plastic bottle and other debris on the axis of Illa Negra branch, with *Echinus* cf. *acutus* (314 m, incised canyon). G: Top of a roman amphora, almost totally buried by sediment at the axis of Illa Negra branch, with unidentified fish (299 m, footwall).

In parallel with cold-water and red coral identification, litter and seafloor disturbance by bottom trawling were accounted during video analysis. Litter was observed in all ROV immersions except for transect 58, which, although being devoid of litter items, is markedly impacted by trawling (S2 Video). In 604 out of a total of 7914 position fixes, a wide variety of litter items was observed resting on the seafloor. This represents 7.6% of the video footage (Fig 6). Litter consists mostly of lost or entangled ropes and longlines (Figs 10A, 10B, S6A, S6B and S6C), nets (Figs 10D, 10E, S6D and S6E) and other fishing gear (71% of litter items), whereas the rest (29%) comprises plastic bags, bottles (Fig 10F), cans, but also other items such as a large plastic box (or an upturned boat, Fig 10F), Roman amphorae (Fig 10G), and a soccer ball. Ropes, longlines and nets often posed a serious risk for ROV navigation (S2 Video).

Litter has been observed at all depths, although it is scarce at less than 100 m water depth (only 2 objects out of 623 position fixes), and at all slope gradients. It is especially abundant at two depth ranges: between 100 and 160 m and between 260 and 300 m water depth (Fig 7). There are less litter items in flatter areas, and are more frequent in topographically elevated places with respect to the surroundings, as indicated by the bathymetric position indexes (Table 6). Litter has mostly been imaged resting on the upper canyon wall (244 items), in narrow slope crests (93) and steep slopes (82), and is proportionally more abundant in narrow deep crests (20% of observations on narrow deep crests included litter), near-vertical walls (17%), upper walls (17%) and shelf crests (15%). On the contrary, gentle slopes (1%) and the incised canyon classes (1%) display less litter (Table 5). Maximum entropy model predicts that 0.55 km^2^ out of 6.94 km^2^ of the modelled area between 75 and 410 m water depth yield probabilities over 70% of being directly impacted by litter (Fig 6D).

Ropes, longlines and nets relate to CWC, and specifically to *M*. *oculata* colonies, in two ways. On one hand, they have been observed entangled in coral patches (Figs 10B, S6A and S6B, S2 Video). Often, accumulations of coral branches have been observed at the foot of colonies damaged by this kind of litter, as imaged in Figs 10C and S6G and S6F, resulting in dead coral rubble deposits (115 position fixes). At specific locations, living branches were imaged detached from the main colony, resting over muddy bottom and close to this kind of litter (S6F Fig), while still acting as small shelters for other fauna such as *Munida* sp. On the other hand, *M*. *oculata* uses specific types of litter as hard substrate on which to attach and grow thus complementing natural substrates such as rocky outcrops, pebbles and cobbles. In La Fonera canyon, some *M*. *oculata* colonies were imaged fixed on ropes and longlines, such as those imaged in S4A Fig.

**Fig 11:**
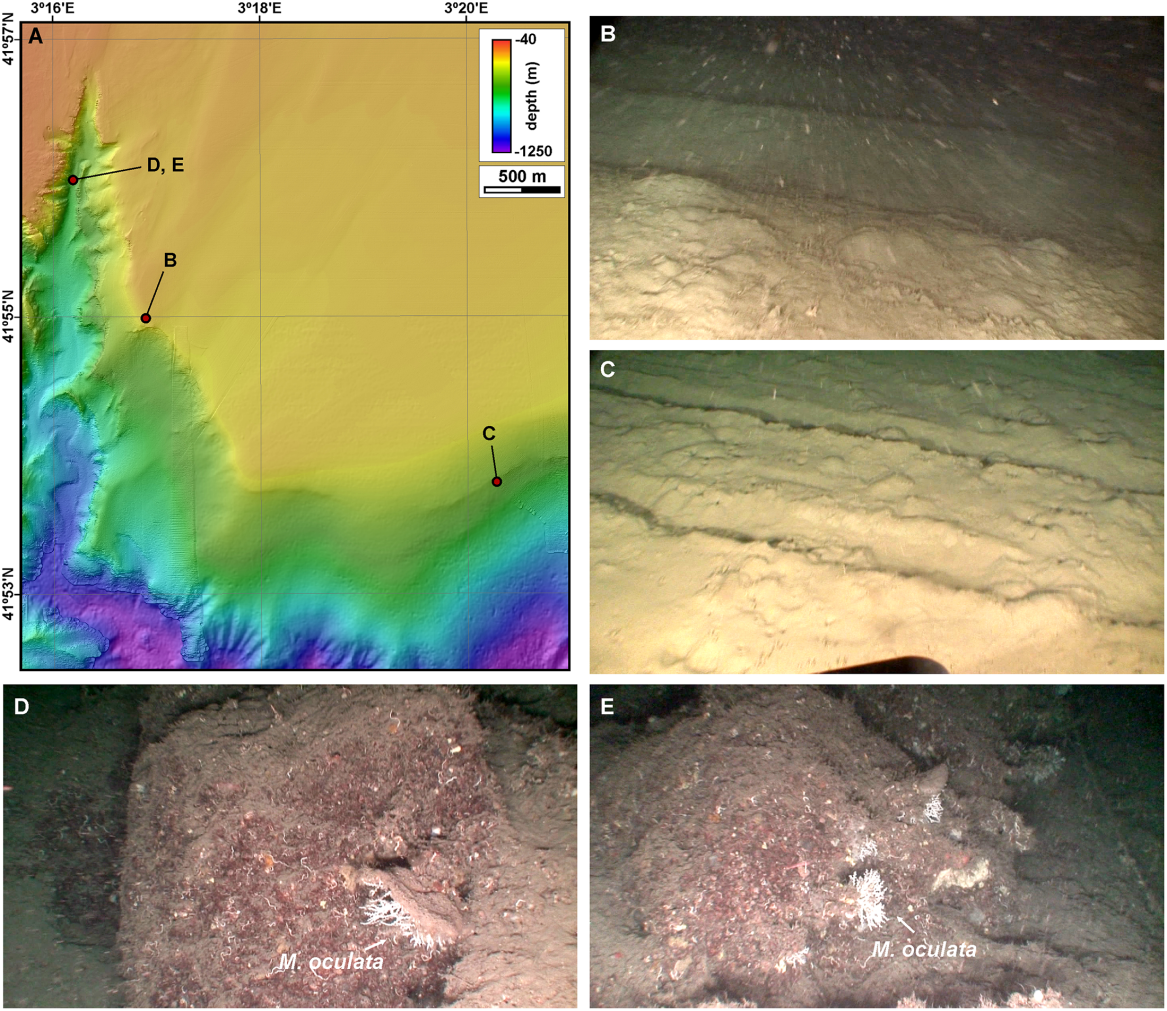
Examples of trawling impacts observed on the seafloor of the study area. A: Locations of ROV images in this figure (B to G). B: Trawling mark within a muddy bottom at the western limit of Sant Sebastià fishing ground (182 m, ramp). C: Trawling mark in the upper eastern La Fonera wall (338 m, gentle slope). D: *M*. *oculata* capped by fine sediment (387 m, footwall). E: *M*. *oculata* partially covered by sediment, with a longline in the background to the right (387 m, footwall).

Trawl marks have been identified at 322 position fixes, mostly in transects located within Sant Sebastià fishing ground (Fig 2). They are imaged as metric-wide linear depressions with overconsolidated mud clasts accumulations on their sides (Fig 11B–11E), among large expanses of the seafloor devoid of life except for scattered *Cerianthus* sp. (S2 Video). In general, those transects close to or within the fishing ground yielded images with an increased amount of suspended particulate material (Fig 11B and 11C). Some coral colonies located on the eastern La Fonera canyon wall have been observed to be partially covered by fine mud, to the extent that coral branches are only partly visible (Fig 11D and 11E and S2 Video).

## Discussion

### CWC communities in La Fonera canyon

The shallow (<260 m) and deep (>260 m) sections of the axis of La Fonera canyon head are mostly covered by sand and finer sediment (Table 5), respectively, and are poorly occupied by benthic communities with erect invertebrates. Canyon walls are rocky and abrupt (Table 5), with abundant caves and overhangs, and host a large variety of benthic fauna. The canyon rim is often covered by sand but also occupied by boulders and outcropping rocks (Table 5), the latter colonised by numerous organisms. Medium-sized colonies of *M*. *oculata* as well as more sparsely distributed *D*. *cornigera* colonies have been imaged in La Fonera canyon head, which allows extending further south the Northwestern Mediterranean CWC province, now including Cassidaigne, Lacaze-Duthiers and Cap de Creus canyons.

In general, larger and healthier *M*. *oculata* colonies in La Fonera canyon are located in the rougher rocky areas of the canyon walls with higher slope gradients. These areas have been classified as near-vertical walls, narrow slope crests and gullies by benthic terrain modelling based on morphological parameters, although the latter have only been sparsely surveyed. Often, these colonies appear in overhangs, as also observed by [23] in the canyons of the Gulf of Lion, and are oriented horizontally or downwards. Smaller *M*. *oculata* colonies have been observed in more isolated blocks and linear escarpments along the canyon walls. Among the areas within La Fonera canyon where CWC have been imaged, the occurrence of *M*. *oculata* on the western wall of Cap de Begur branch, at depths not exceeding 150 m, is among the shallowest locations in which CWC have been found, together with the Strait of Gibraltar and Cassidaigne canyon [15, 23].

On the contrary, *D*. *cornigera* preferentially settles on moderately sloping areas, which have been classified as steep slopes. Red coral *C*. *rubrum* colonies prefer shallower waters (<160 m) and thus are present on the upper but also highly sloping sections of the canyon walls, classified as upper walls and near-vertical walls. Studies conducted in front of Cap de Creus promontory between 50 and 230 m depth showed that red coral is less abundant below 120 m water depth, where it has a more scattered distribution with isolated colonies, similar to our observations in La Fonera canyon, in contrast to dense patches observed in shallower water (50–85 m) [34]. Deep (>200 m) occurrences of *C*. *rubrum* were also recorded, in coexistence with *M*. *oculata*. Co-occurrence of red coral with scleractinian corals *L*. *pertusa* and *M*. *oculata* has been reported at depths ranging between 200 and 460 m in the Gulf of Lion [23] and the Sicily Channel [19, 30].

A characteristic of the CWC community in La Fonera canyon is the absence of *L*. *pertusa*, in contrast with Cap de Creus and Lacaze-Duthiers canyons [20, 23, 35], at least within the depth range investigated. Although this species is scarce and in decline in the Mediterranean [16, 24], its absence in the shallow reaches of the canyon, together with the dominance of middle-sized, fragile *M*. *oculata* colonies, the poor development of the small-sized *D*. *cornigera* colonies, and the lack or low occurrence of gorgonians and other usual species of these communities may be indicative that La Fonera CWC community is close to the environmental limits in which these species can develop, and /or withstands a high environmental stress. Among the three species, *M*. *oculata* presents higher growth rates and is better adapted to such stressing environments [55].

### Favourable environments for CWC communities in La Fonera canyon

Morphological parameters derived from bathymetric data, such as slope, seafloor roughness, topographic indexes, curvature and slope directions, are proven surrogates for sedimentary environments, and are commonly used in multivariable classifications of the seabed and species distribution modelling [56, 57, 58]. [57] evidenced how the use of high-resolution multibeam bathymetry, in their case obtained with a ROV-mounted multibeam system in the Porcupine Seabight, yielding a 0.5 m bathymetric grid cell size at 800–900 m water depth, allowed resolving bathymetric details that are relevant in structuring the distribution of CWC. In our case, a hull-mounted multibeam system operated in equidistant mode was able to resolve such details at depths not exceeding 400 m. These parameters are indeed related to the factors affecting the distribution of CWC. This relation is quite direct regarding coral-suitable substratum, since hard substrates will most probably occur on high sloping areas, often with relatively high roughness, whereas favourable water-mass properties and food availability will be related with water depth and current patterns, the later partially controlled by seabed topography.

Based on these parameters, maximum entropy modelling allows inferring the possible extension of CWC suitable habitats in La Fonera canyon. Nevertheless, modelling results should be taken with caution since the distribution of ROV observations in the study area does not represent equitably the different types of terrains. For example, ROV surveying effort in the outer shelf and canyon floor is nil, and the ramp only accounts for 0.6% of all observations although representing 29.1% of the area (Tables 3 and 4). In addition, ROV observations are constrained to a given depth interval (deeper than 79 m, shallower than 401 m) and locations outside that interval lack observations. Therefore, beyond these water depths maximum entropy predictions are unfounded. In addition, maximum entropy prediction of habitat suitability only takes into account positive observations.

Nevertheless, not all favourable grounds identified by modelling are indeed occupied by CWC. Large sections of transects located along areas where corals are predicted to occur did not show evidence of coral presence, and no large and healthy colonies similar to those observed in the eastern wall of Cap de Begur branch were observed in Illa Negra branch. This could be related to the model lacking a key environmental parameter that ultimately determines coral presence or absence when topographic conditions define a suitable habitat. Such environmental parameter could be related to food availability, sediment concentration and/or hydrodynamics.

One common characteristic of the canyons conforming the Northwestern Mediterranean CWC province is that they have their heads incised in the continental shelf, especially Cap de Creus and La Fonera canyons, whose heads are located at short distances from the coastline. This favours capturing dense shelf water when generated [38, 45, 46] and probably also sediment-laden waters of coastal origin during eastern storms as it happens in Blanes canyon further south [59], enhancing hydrodynamics and food arrival. Thus, canyon morphology as well as meteorology and oceanography configure particularly favouring conditions that determine the extent of this CWC province, even though water temperature is generally slightly beyond the optimal conditions for CWC development [60]. As a counterpart, this configuration can also involve an excess of sediment arrival. Thus, CWC do not occupy all morphologically favourable grounds throughout these canyons, but only specific locations where hydrodynamics prevent high sedimentation rates [22]. At these locations, hard substrates are sediment-free and ready for larval settlement.

[19] suggested that canyon systems might better facilitate the extent of coral assemblages into shallower depths than open slopes due to seasonal or episodic cascading of water masses loaded with nutrients, food, and organic matter. CWC prefer highly energetic hydrodynamic environments, but although Illa Negra branch presents high current speeds (>50 cm·s^-1^) during dense shelf water cascading events triggered or enhanced by storms [46], corals are scarce in this branch compared to Cap de Begur branch. [20] related CWC communities in Cap de Creus canyon to the energetic dense shelf water cascading flows carrying periodically nutritive particles in suspension from the shelf environments and to the reduced sediment accumulation rates caused by this process, but this seems not to be the case of Illa Negra branch where cascading events are likely milder. On the contrary, the proximity to the coastline, with the Illa Negra branch tip at a distance of barely 800 m from shore, could promote the arrival of an excess of sediment intercepted from the coastal sediment drift during storms with detrimental effects on coral development. Note, for instance, the thin sediment veneer covering rock outcrops in S1A, S1C, S4C and S4D Figs compared to rock outcrops in Cap de Begur branch (Fig 5B and 5C). This branch would be, conversely, more protected from the arrival of excess sediment but still within the effects of high energetic processes, similarly to the more distant sections of Illa Negra branch where corals grow (Fig 8E).

### Litter in La Fonera canyon and interaction with CWC

La Fonera canyon has been reported to host one of the largest mean concentrations of litter on the deep-sea floor [51]. Although [51] concluded that canyon walls seem to be relatively less impacted than the canyon floor in terms of number and density of litter items, our observations indicate that within the inspected depth range of 79 to 401 m, litter is specially abundant on the canyon walls and crests, whereas is relatively less abundant along the floor. Objects in the canyon walls are mostly linear-shaped items entangled in rock outcrops, such as ropes, longlines and nets; whereas litter in the canyon floor are round-shaped items more easily transported such as light plastics, bottles or cans. High energetic events such as storms and cascading, which are most probably the key elements that favour the extension of the Northwestern Mediterranean CWC province, also favour the transport of light and round-shaped items down the walls to the canyon floor, and then down-canyon, and concentrate them along the canyon floor surveyed by [51]. On the other hand, those linear-shaped objects that can easily be entangled, generally lost and abandoned fishing gear, and other heavy litter items, simply stay close to their original locations in the upper canyon walls, where fishing effort is larger.

Out of a total of 497 position fixes where any of the three species studied has been observed, litter is observed in the same field of view in 116 locations (23.3%). In general terms, the relation between CWC and litter presence is negative, with ropes, longlines and nets observed to be entangled and damaging coral colonies. Less protected areas and isolated colonies are highly vulnerable and much more impacted by this kind of litter, for example those located in the western limit of the Sant Sebastià fishing ground (Fig 2). Similar impacts to those observed in La Fonera canyon (entangled nets, coral rubble) have also been described in the neighbouring Cap de Creus canyon, where on average one fishing line was found every 10 m of video transect, often entangled on coral colonies [61].

### Trawling impact

Impact of bottom trawling has previously been documented in many CWC communities such as those located west of Ireland and offshore Norway [62, 63, 64] and in the Northwestern Mediterranean Sea [20, 23]. In La Fonera canyon, high sloping, rocky areas where the largest colonies have been imaged are less prone to be directly impacted (i.e. breaking and crushing) by trawling because they are already known as dangerous areas for the nets and avoided by local fishermen. In a way, the rougher and harder the terrain, the more self-protective against trawling it is.

In addition to direct impact by trawling, frequent bottom trawl activity alters the hydrodynamic and sedimentary conditions, particularly increasing suspension of sediment, further impacting suspension feeders such as coral species [2]. [65] demonstrated that although *L*. *pertusa* can tolerate fairly high sedimentation, mortality increases rapidly with extreme or persistent suspended sediment exposure even if food is available. [66] reported that the skeletal growth of this coral species is significantly lower under sediment concentrations of 25 mg·l^−1^. In La Fonera canyon area, bottom trawling along the Sant Sebastià fishing ground at water depths ranging between 170 and 750 m or deeper generates daily sediment transport events with concentrations of up to 236 mg·l^−1^ close to the bottom [43, 48] and contributes to the development of slope nepheloid layers, propagating suspended sediment away from fishing grounds [50]. This way, human activities add stress to a naturally stressing environment. Coral colonies partially suffocated by fine mud on the eastern La Fonera canyon wall could be the result of increased sediment settling due to trawling, in excess to the capacity of *M*. *oculata* in shedding it through ciliary action and mucus production where local currents are not strong enough to clean it away.

Anthropogenic impacts such as the presence of lost and abandoned fishing gear and both the direct and indirect impact of trawling may be nowadays intrinsic to CWC provinces since the conditions that control CWC growth as well as their role as other species’ refuges for prey and nursery areas determine their high biodiversity and thus their appeal as fishing spots. The European Marine Strategy Framework Directive (MSFD) established in 2008 aims at assessing and reducing direct and indirect human impacts on marine ecosystems of Europe, with the objective of maintaining or achieving good environmental status by 2020. The General Fisheries Commission for the Mediterranean (GFCM) identified in 2008 sensitive habitats of relevance for the management of priority species that are potentially vulnerable, which included CWC *L*. *pertusa* and *M*. *oculata* communities. In this regard, the observed impacts of trawling on CWC in La Fonera canyon should be a wakeup call for increasing the effort put on studying the distribution of these species in submarine canyons of the Mediterranean Sea and controlling fishing activities in the areas where pristine colonies can still be found.

## Conclusions

Colonies of CWC *M*. *oculata* and *D*. *cornigera* have been observed in La Fonera canyon head, in the Northwestern Mediterranean Sea, by means of ROV video imaging. These positive identifications extend further west and south previous observations in submarine canyons of the Gulf of Lions, particularly Cassidaigne, Lacaze-Duthiers and Cap de Creus canyons, thus defining an extensive CWC province in the Mediterranean Basin. *M*. *oculata* was imaged in 12 out of a total of 21 transects and *D*. *cornigera* in 14. *M*. *oculata* forms, on the walls of Cap de Begur branch, dense middle-sized healthy colony clusters hosting a wide variety of fauna, whereas in other locations within the canyon head colonies are relatively smaller and more isolated. *D*. *cornigera* colonies are small-sized and isolated. Red coral *C*. *rubrum* also is present in La Fonera canyon head, where it has been imaged in 10 ROV transects. *L*. *pertusa* has not been observed in any of the transects.

Benthic terrain modelling based on swath bathymetry data has allowed classifying the different environments of the canyon head, and identifying in which terrains the different species concentrate. *M*. *oculata* and *C*. *rubrum* grow mostly on rocky near-vertical walls, the later at shallower water depths, whereas *D*. *cornigera* prefers sedimented or rocky steep slopes. The three species have been observed to live together. Maximum entropy prediction of habitat suitability indicates that out of a study area of 6.94 km^2^ between 75 and 410 m water depth, 0.36 km^2^ yield probabilities over 70% of occurrence of *M*. *oculata*, 0.43 km^2^ of *D*. *cornigera* and 0.15 km^2^ of *C*. *rubrum*. Nevertheless, due to hydrodynamics and other conditions beyond canyon morphology, not all suitable modelled habitats are occupied by CWC. Particularly, corals are much scarcer in Illa Negra branch than in Cap de Begur branch, the former incised much closer to the coastline.

Litter items resting on the seafloor have been observed throughout the canyon head whereas direct impact by bottom trawling is evident on the upper eastern wall. Fishing items such as ropes, nets and longlines are entangled on CWC colonies, and detached coral fragments and coral rubble have been imaged at the foot of damaged colonies. Other colonies are partially covered by fine sediment, possibly as a result of enhanced sediment fluxes due to daily resuspension by bottom trawling. The characteristics of the CWC community in La Fonera canyon are indicative that it withstands a high environmental stress, of both natural and human origin. This study represents the first step of a necessary assessment of the distribution of CWC along one of the largest submarine canyons in the entire Mediterranean Sea and highlights the need of identifying and mitigating human impacts to these unique habitats.

## Supporting Information

S1 Fig ROV images depicting substrate types in the study area. Location in Fig 1. A: Rock outcrops covered by a very thin mud veneer at the foot of Illa Negra western wall (306 mwd, footwall). B: Rock outcrop in the western wall of Illa Negra branch (246 mwd, near-vertical wall), with sponges *Poecillastra compressa*, *Hamacantha* cf *falcula*, polychaete *Vermiliopsis* sp. and brachiopod *Gryphus vitreus*. C: Rock blocks covered by a thin mud veneer at the foot of Illa Negra eastern wall (285 mwd, footwall), with brachiopod *G*. *vitreus*. D: Transition between a muddy bottom at the axis of Cap de Begur branch and a rock outcrop (373 mwd, near-vertical wall). E: Transition between a bioclastic sandy bottom and rock outcrops on the eastern canyon rim close to Sant Sebastià fishing ground (183 mwd, steep slope), with *D*. *cornigera* in the background. F: Bioclastic sand along the axis of Illa Negra branch (215 mwd, incised canyon). G: Sandy bottom on the eastern wall of Illa Negra branch (116 mwd, upper wall). H: Sandy bottom on the eastern rim of Cap de Begur branch (154 mwd, canyon rim), with *Scyliorhinus canicula*.
(TIF)

S2 Fig ROV images depicting substrate types in the study area. Location in Fig 1. A: Bioclastic sand on the eastern wall of the canyon close to Sant Sebastià fishing ground (226 mwd, gentle slope), with *Octopus salutii* and hydrarians. B: Muddy bottom along the axis of Cap de Begur branch with *L*. *conchilega* (387 mwd, footwall). C: Muddy bottom along the axis of Illa Negra branch (280 mwd, incised canyon), with *Molva dipterygia*. D: Muddy bottom on the eastern wall of the canyon close to Sant Sebastià fishing ground with trail marks (353 mwd, steep slope). E: Muddy bottom on the eastern wall of the canyon close to Sant Sebastià fishing ground (315 mwd, gentle slope), with *O*. *salutii*. F: Muddy bottom on the eastern wall of the canyon close to Sant Sebastià fishing ground (275 mwd, gentle slope), with tube-dwelling anemone *Cerianthus membranaceus* and decapod *Pagurus prideaux*. G: Muddy bottom on the inner shelf, south of Illa Negra branch (98 mwd, inner shelf), with soft coral *Alcyonium palmatum*. H: Muddy bottom with bioclasts on the eastern rim of Cap de Begur branch (190 mwd, gentle slope).
(TIF)

S3 Fig ROV images depicting cold-water and red coral colonies in the study area. Location in Fig 1. A: Dense overhanging *M*. *oculata* living colonies at the foot of Cap de Begur eastern wall (205 m, near-vertical wall). B: *M*. *oculata* and *D*. *cornigera* living colonies at the foot of Cap de Begur eastern wall (236 m, footwall). C: *M*. *oculata* and *D*. *cornigera* living colonies at the upper part of Cap de Begur eastern wall, with decapod *P*. *elephas* (157 m, upper wall). D: *M*. *oculata* living colonies on a relatively flat area on the rim of the western wall of Cap de Begur branch (131 m, shelf crest). E: Detail of *M*. *oculata* living colony with polychaete *Salmacina dysteri*, paguroid *Dardanus arrosor* and anemone *Calliactis parasitica* on top, on the western wall of Cap de Begur branch (223 m, gully). F: Downward growing *M*. *oculata* living colonies on the western wall of Cap de Begur branch, with their upper sides partly covered by mud, with brachiopod *G*. *vitreus* and decapod *Munida* sp. (243 m, near-vertical wall). G: *M*. *oculata* and *C*. *rubrum* living colonies with decapod *Munida* sp., polychaetes *P*. *tubularia* and *S*. *pavonina*, bryozoan *Reteporella* sp. and demosponge *Crella* cf. *pulvinar* on the upper part of the eastern La Fonera canyon head wall (265 m, steep slope), H: *M*. *oculata*, *D*. *cornigera* and *C*. *rubrum* living colonies and solitary coral *Caryophyllia* sp. on the upper part of the eastern La Fonera canyon head wall, close to Sant Sebastià fishing ground, with brachiopod *G*. *vitreus* (222 m, narrow slope crest).
(TIF)

S4 Fig ROV images depicting cold-water and red coral colonies in the study area. Location in Fig 1. A: Living colony of *M*. *oculata* growing on lost fishing gear, with ophiurioid *Ophiothrix* sp., polychaetes *S*. *pavonina* and *Vermiliopsis* sp., oyster *N*. *cochlear* and sponge *Reniera* sp. (279 m, gentle slope). B: *D*. *cornigera* isolated polyp, together with sponge *Axinella damicornis* and small colony of *C*. *rubrum* on the eastern wall of Illa Negra branch (159 m, upper wall). C: *D*. *cornigera* colony and solitary corals *Caryophyllia* sp. on the eastern wall of Illa Negra branch (233 m, steep slope). D: *D*. *cornigera* colony on the eastern wall of Illa Negra branch (248 m, divide). E: *D*. *cornigera* colony on the western wall of Illa Negra branch (247 m, incised canyon). F: *D*. *cornigera* colony on the western wall of Cap de Begur branch (159 m, upper canyon). G: *C*. *rubrum* colonies on the roof of a cave on Sant Sebastià branch (184 m, near-vertical wall). H: *C*. *rubrum* colonies in an overhanging rock on Sant Sebastià branch (115 m, upper wall).
(TIF)

S5 Fig Jackknife tests of variable importance. For each plot, red bar indicates the total gain when all variables are used; blue bars indicate the gain when each environmental variable is used in isolation, the highest gains indicating the variables that appear to have the most useful information by themselves; and green bars indicate the gain when the environmental variable is omitted, therefore the lowest gains indicating the variables that appear to have the most information that is not present in the other variables.
(TIF)

S6 Fig Examples of litter observed on the seafloor of the study area. Location in Fig 1. A: Longline entangled on *M*. *oculata* colonies located on a rock outcrop near the axis of Cap de Begur branch (369 m, near-vertical wall). B: Longlines and ropes entangled on the rocks of the western wall of Illa Negra branch, with *M*. *oculata* colonies (194 m, steep slope). C: Rope located at the foot of a rock outcrop where CWC grow near the axis of Cap de Begur branch (387 m, footwall). D: Fishing nets entangled on rock outcrop near the western wall of Cap de Begur branch, with oysters *N*. *cochlear* (270 m, incised canyon). E: Fishing net entangled on a rock outcrop on the eastern wall of Illa Negra branch, with sea cucumber *Holothuria forskali* (126 m, upper wall). F: Detached but still living *M*. *oculata* colony resting next to a longline at the foot of the eastern wall of Cap de Begur branch, next to large colonies imaged in Fig 8B and 8C (289 m, incised canyon). G: Dead *M*. *oculata* coral rubble at the foot of the western wall of Illa Negra branch, next to large colonies imaged in Fig 10B (223 m, near-vertical wall). H: Dead coral rubble on the upper part of the eastern La Fonera canyon wall, next to colonies imaged in S3G, S3H and S4A Figs (224 m, narrow slope crest).
(TIF)

S1 Spreadsheet Compressed file including spreadsheet with all observations and bathymetric-derived parameters, and text file with instructions. (ZIP)

S1 Video Examples of cold-water and red coral colonies in the study area. Most examples image the largest colonies in Cap de Begur branch (transects 16, 17, 20 and 22) as well as the only large one observed in Illa Negra branch (transect 9). Other occurrences in the canyon are smaller in size. Note also that some images example dead coral rubble in the vicinity of entangled longlines.
(AVI)

S2 Video Examples of human impacts in the study area. These include litter (plastics and other garbage, and entangled nets and longlines), increased sediment loads and trawling marks.
(AVI)
