# Supplementary figures and images for: Cold-Water Corals and Anthropogenic Impacts in La Fonera Submarine Canyon Head, Northwestern Mediterranean Sea
Source: PLoS One. 2016 May 16;11(5):e0155729. doi: 10.1371/journal.pone.0155729 (PMC4868382; doi:10.1371/journal.pone.0155729)

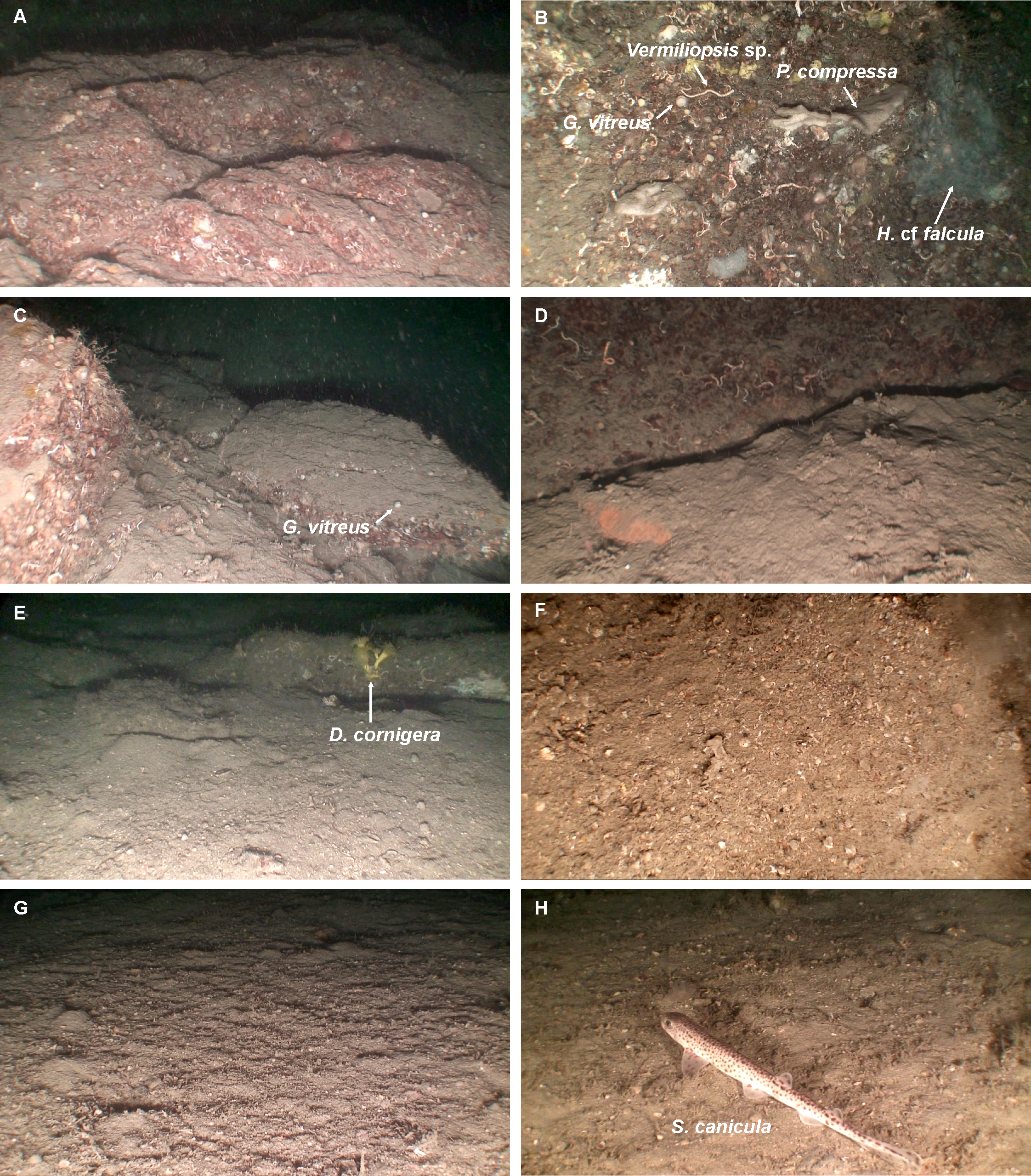

Supplement: S1 Fig — Location in Fig 1. A: Rock outcrops covered by a very thin mud veneer at the foot of Illa Negra western wall (306 mwd, footwall). B: Rock outcrop in the western wall of Illa Negra branch (246 mwd, near-vertical wall), with sponges Poecillastra compressa, Hamacantha cf falcula, polychaete Vermiliopsis sp. and brachiopod Gryphus vitreus. C: Rock blocks covered by a thin mud veneer at the foot of Illa Negra eastern wall (285 mwd, footwall), with brachiopod G. vitreus. D: Transition between a muddy bottom at the axis of Cap de Begur branch and a rock outcrop (373 mwd, near-vertical wall). E: Transition between a bioclastic sandy bottom and rock outcrops on the eastern canyon rim close to Sant Sebastià fishing ground (183 mwd, steep slope), with D. cornigera in the background. F: Bioclastic sand along the axis of Illa Negra branch (215 mwd, incised canyon). G: Sandy bottom on the eastern wall of Illa Negra branch (116 mwd, upper wall). H: Sandy bottom on the eastern rim of Cap de Begur branch (154 mwd, canyon rim), with Scyliorhinus canicula. (TIF) [file pone.0155729.s001.tif]

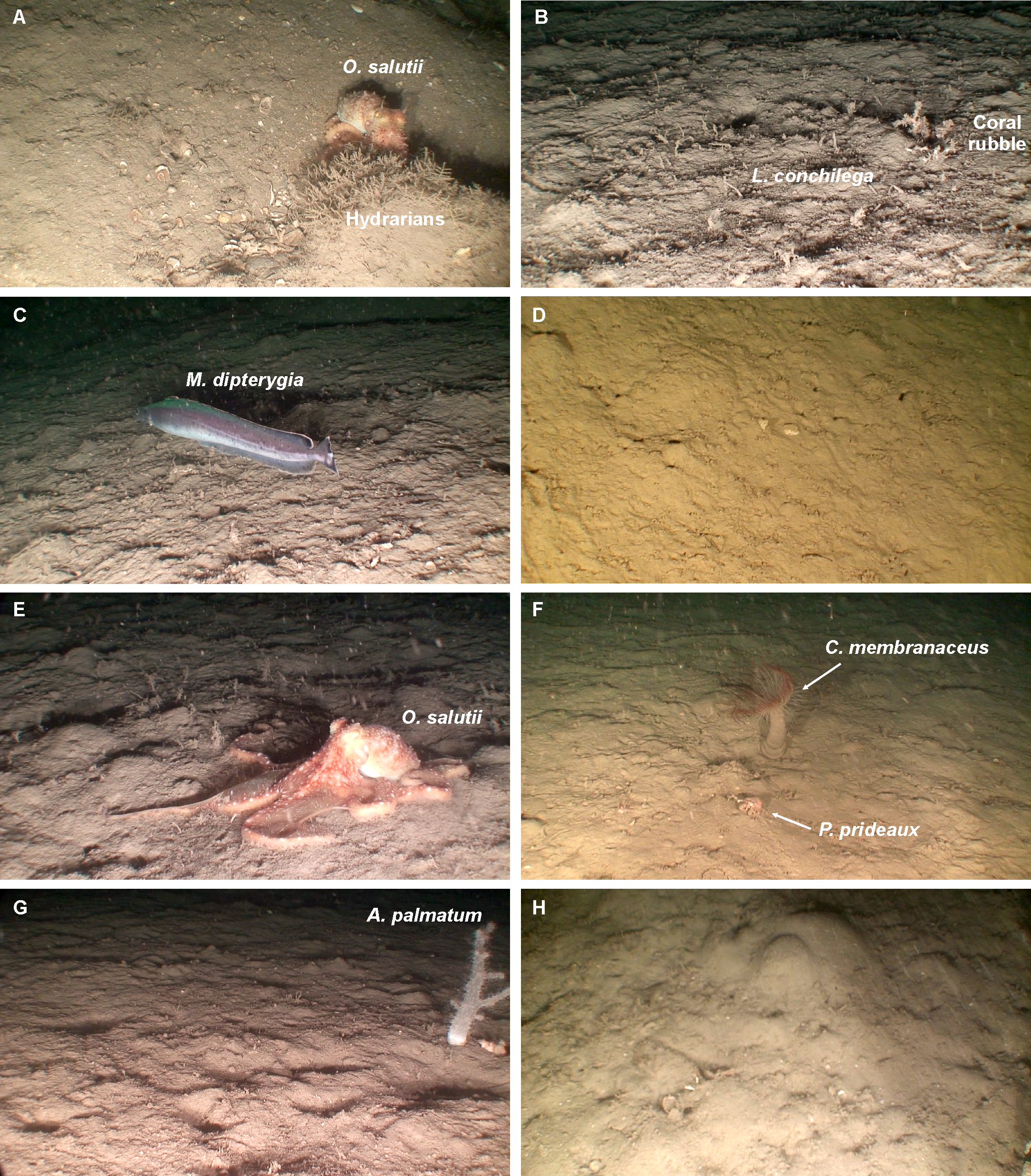

Supplement: S2 Fig — Location in Fig 1. A: Bioclastic sand on the eastern wall of the canyon close to Sant Sebastià fishing ground (226 mwd, gentle slope), with Octopus salutii and hydrarians. B: Muddy bottom along the axis of Cap de Begur branch with L. conchilega (387 mwd, footwall). C: Muddy bottom along the axis of Illa Negra branch (280 mwd, incised canyon), with Molva dipterygia. D: Muddy bottom on the eastern wall of the canyon close to Sant Sebastià fishing ground with trail marks (353 mwd, steep slope). E: Muddy bottom on the eastern wall of the canyon close to Sant Sebastià fishing ground (315 mwd, gentle slope), with O. salutii. F: Muddy bottom on the eastern wall of the canyon close to Sant Sebastià fishing ground (275 mwd, gentle slope), with tube-dwelling anemone Cerianthus membranaceus and decapod Pagurus prideaux. G: Muddy bottom on the inner shelf, south of Illa Negra branch (98 mwd, inner shelf), with soft coral Alcyonium palmatum. H: Muddy bottom with bioclasts on the eastern rim of Cap de Begur branch (190 mwd, gentle slope). (TIF) [file pone.0155729.s002.tif]

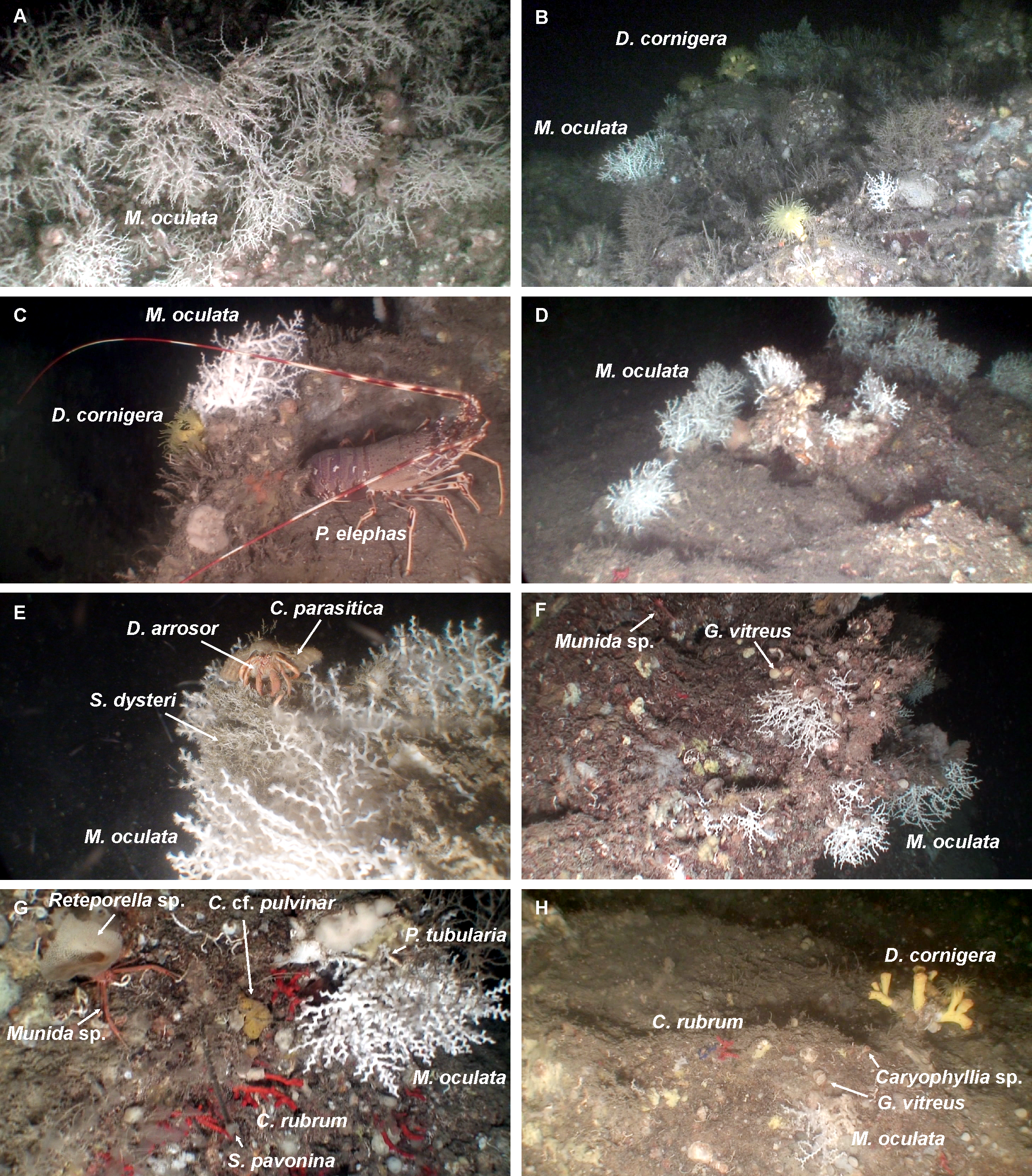

Supplement: S3 Fig — Location in Fig 1. A: Dense overhanging M. oculata living colonies at the foot of Cap de Begur eastern wall (205 m, near-vertical wall). B: M. oculata and D. cornigera living colonies at the foot of Cap de Begur eastern wall (236 m, footwall). C: M. oculata and D. cornigera living colonies at the upper part of Cap de Begur eastern wall, with decapod P. elephas (157 m, upper wall). D: M. oculata living colonies on a relatively flat area on the rim of the western wall of Cap de Begur branch (131 m, shelf crest). E: Detail of M. oculata living colony with polychaete Salmacina dysteri, paguroid Dardanus arrosor and anemone Calliactis parasitica on top, on the western wall of Cap de Begur branch (223 m, gully). F: Downward growing M. oculata living colonies on the western wall of Cap de Begur branch, with their upper sides partly covered by mud, with brachiopod G. vitreus and decapod Munida sp. (243 m, near-vertical wall). G: M. oculata and C. rubrum living colonies with decapod Munida sp., polychaetes P. tubularia and S. pavonina, bryozoan Reteporella sp. and demosponge Crella cf. pulvinar on the upper part of the eastern La Fonera canyon head wall (265 m, steep slope), H: M. oculata, D. cornigera and C. rubrum living colonies and solitary coral Caryophyllia sp. on the upper part of the eastern La Fonera canyon head wall, close to Sant Sebastià fishing ground, with brachiopod G. vitreus (222 m, narrow slope crest). (TIF) [file pone.0155729.s003.tif]

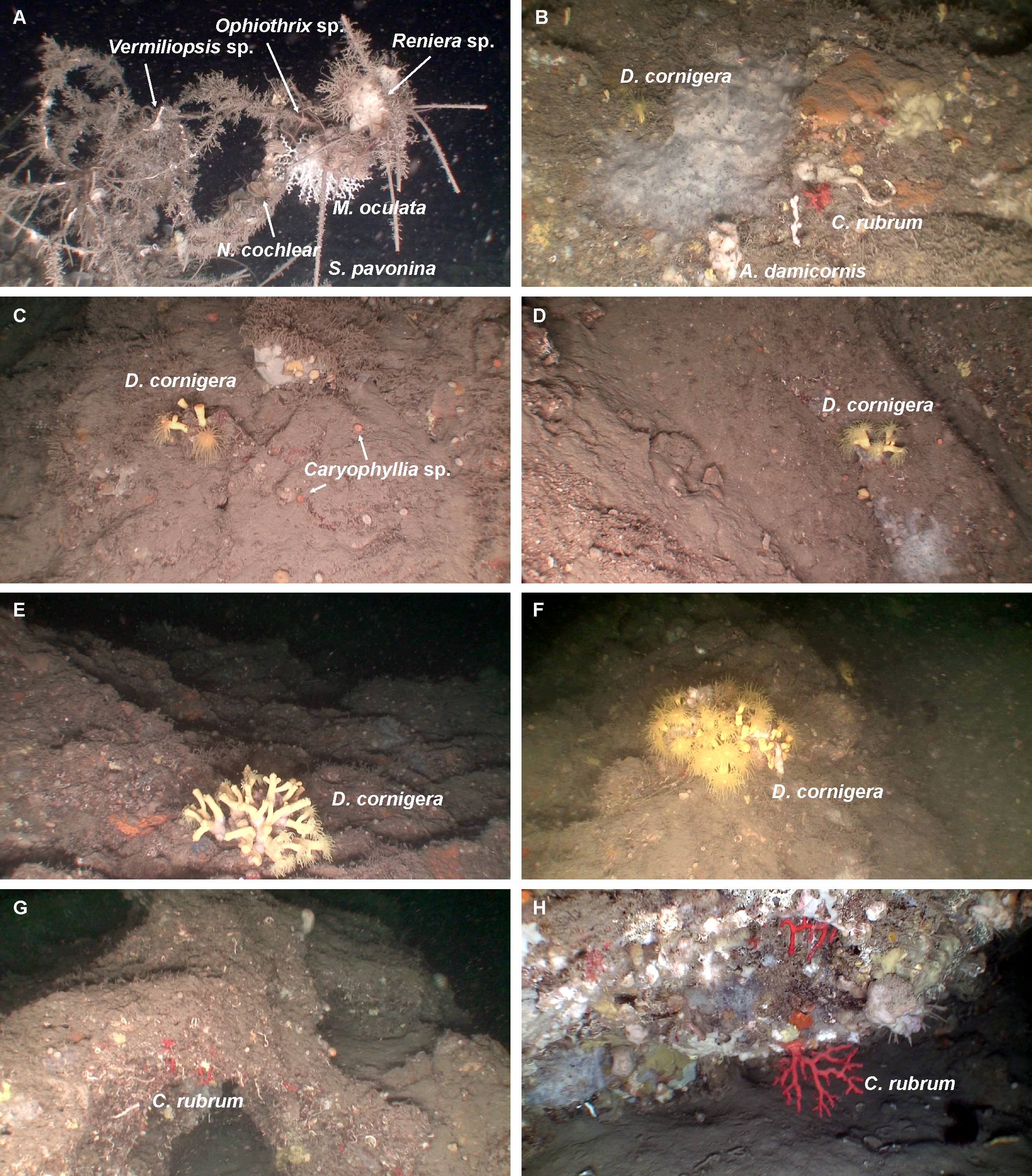

Supplement: S4 Fig — Location in Fig 1. A: Living colony of M. oculata growing on lost fishing gear, with ophiurioid Ophiothrix sp., polychaetes S. pavonina and Vermiliopsis sp., oyster N. cochlear and sponge Reniera sp. (279 m, gentle slope). B: D. cornigera isolated polyp, together with sponge Axinella damicornis and small colony of C. rubrum on the eastern wall of Illa Negra branch (159 m, upper wall). C: D. cornigera colony and solitary corals Caryophyllia sp. on the eastern wall of Illa Negra branch (233 m, steep slope). D: D. cornigera colony on the eastern wall of Illa Negra branch (248 m, divide). E: D. cornigera colony on the western wall of Illa Negra branch (247 m, incised canyon). F: D. cornigera colony on the western wall of Cap de Begur branch (159 m, upper canyon). G: C. rubrum colonies on the roof of a cave on Sant Sebastià branch (184 m, near-vertical wall). H: C. rubrum colonies in an overhanging rock on Sant Sebastià branch (115 m, upper wall). (TIF) [file pone.0155729.s004.tif]

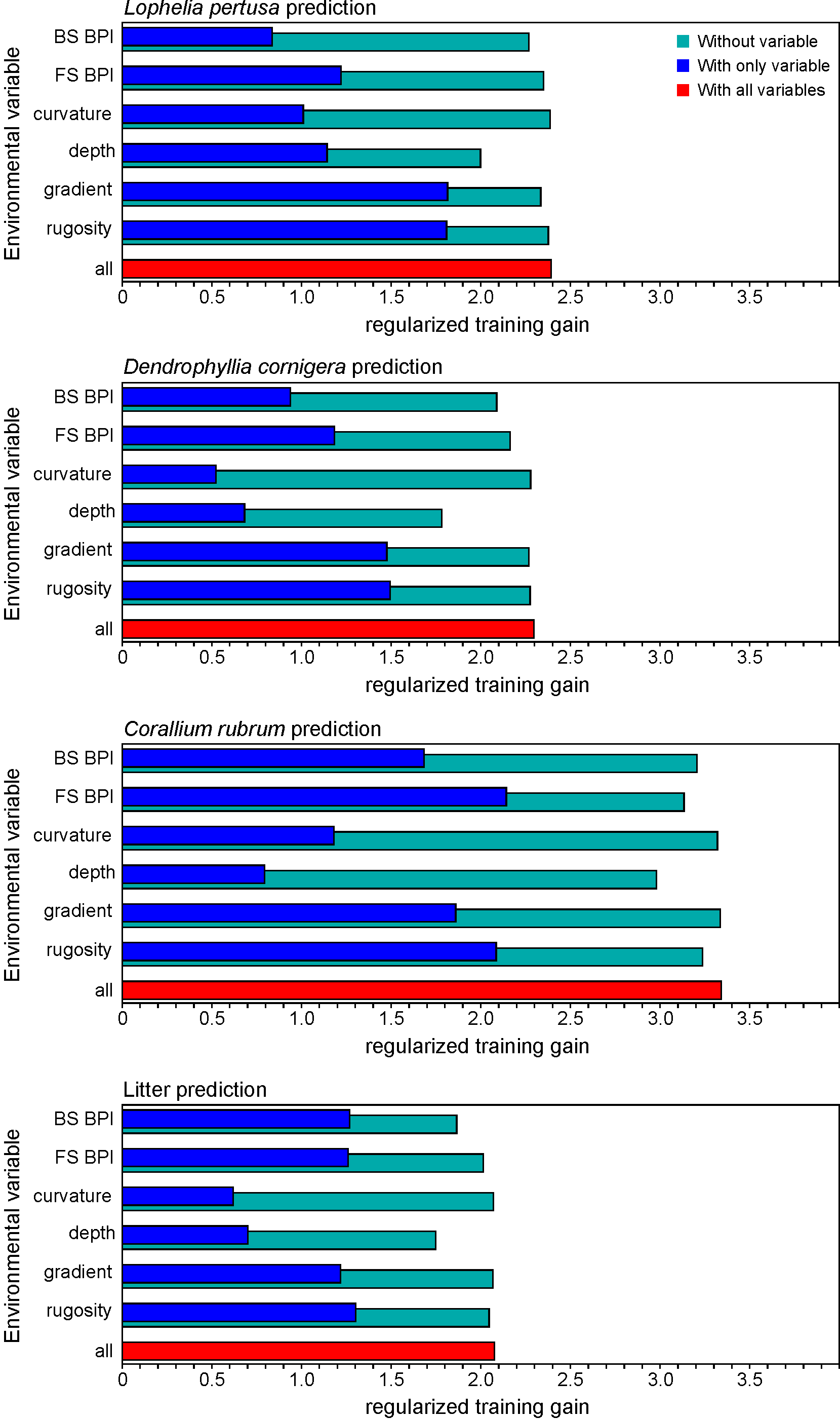

Supplement: S5 Fig — For each plot, red bar indicates the total gain when all variables are used; blue bars indicate the gain when each environmental variable is used in isolation, the highest gains indicating the variables that appear to have the most useful information by themselves; and green bars indicate the gain when the environmental variable is omitted, therefore the lowest gains indicating the variables that appear to have the most information that is not present in the other variables. (TIF) [file pone.0155729.s005.tif]

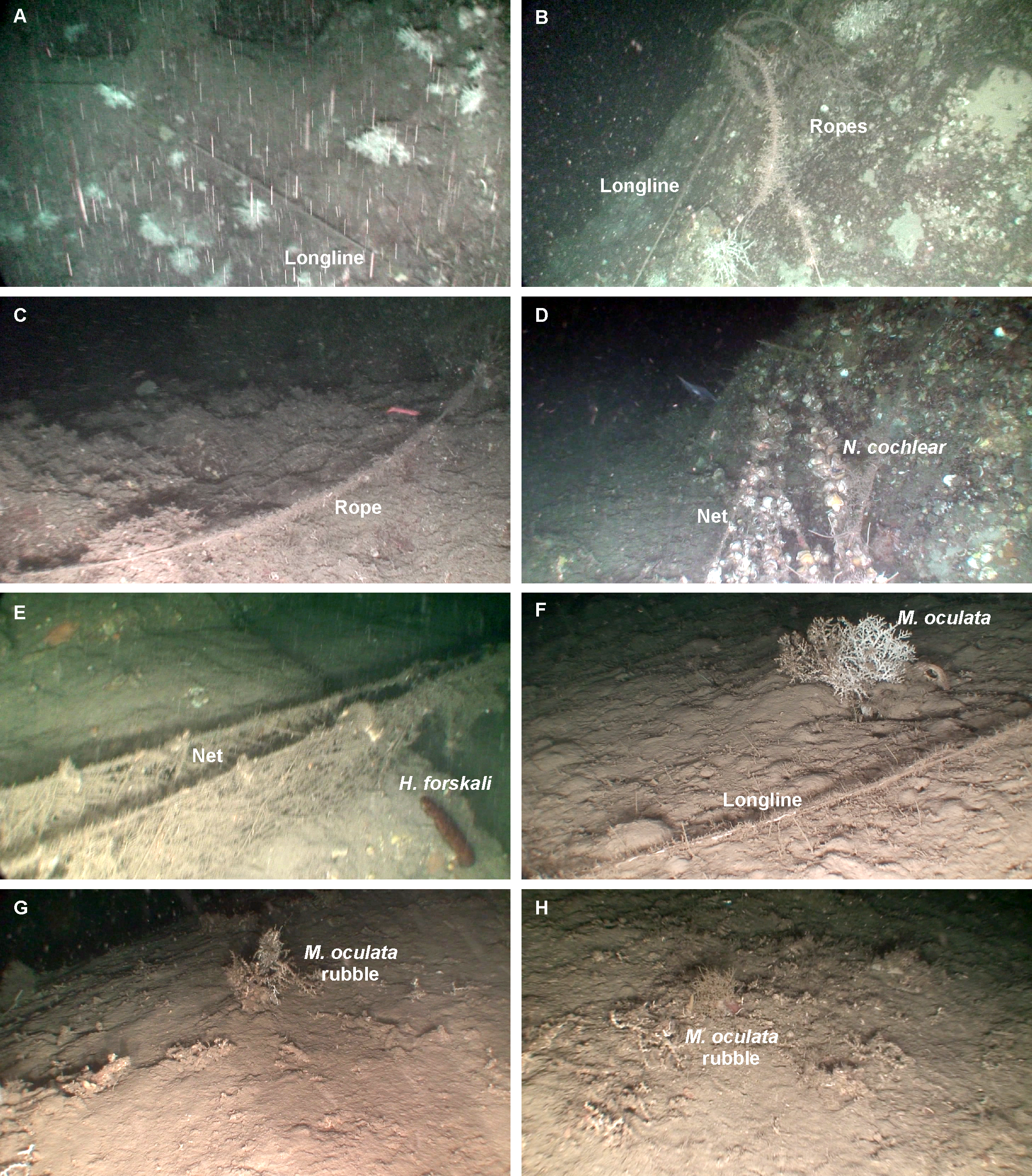

Supplement: S6 Fig — Location in Fig 1. A: Longline entangled on M. oculata colonies located on a rock outcrop near the axis of Cap de Begur branch (369 m, near-vertical wall). B: Longlines and ropes entangled on the rocks of the western wall of Illa Negra branch, with M. oculata colonies (194 m, steep slope). C: Rope located at the foot of a rock outcrop where CWC grow near the axis of Cap de Begur branch (387 m, footwall). D: Fishing nets entangled on rock outcrop near the western wall of Cap de Begur branch, with oysters N. cochlear (270 m, incised canyon). E: Fishing net entangled on a rock outcrop on the eastern wall of Illa Negra branch, with sea cucumber Holothuria forskali (126 m, upper wall). F: Detached but still living M. oculata colony resting next to a longline at the foot of the eastern wall of Cap de Begur branch, next to large colonies imaged in Fig 8B and 8C (289 m, incised canyon). G: Dead M. oculata coral rubble at the foot of the western wall of Illa Negra branch, next to large colonies imaged in Fig 10B (223 m, near-vertical wall). H: Dead coral rubble on the upper part of the eastern La Fonera canyon wall, next to colonies imaged in S3G, S3H and S4A Figs (224 m, narrow slope crest). (TIF) [file pone.0155729.s006.tif]
